# Autism-linked Cullin3 germline haploinsufficiency impacts cytoskeletal dynamics and cortical neurogenesis through RhoA signaling

**DOI:** 10.1038/s41380-021-01052-x

**Published:** 2021-03-16

**Authors:** Megha Amar, Akula Bala Pramod, Nam-Kyung Yu, Victor Munive Herrera, Lily R. Qiu, Patricia Moran-Losada, Pan Zhang, Cleber A. Trujillo, Jacob Ellegood, Jorge Urresti, Kevin Chau, Jolene Diedrich, Jiaye Chen, Jessica Gutierrez, Jonathan Sebat, Dhakshin Ramanathan, Jason P. Lerch, John R. Yates, Alysson R. Muotri, Lilia M. Iakoucheva

**Affiliations:** 1 Department of Psychiatry, University of California San Diego, La Jolla, CA USA; 2 Department of Molecular Medicine, The Scripps Research Institute, La Jolla, CA USA; 3 Mouse Imaging Centre (MICe), Hospital for Sick Children, Toronto, ON Canada; 4 Wellcome Centre for Integrative Neuroimaging, FMRIB, Nuffield Department of Clinical Neuroscience, The University of Oxford, Oxford, UK; 5 Department of Cellular & Molecular Medicine, University of California San Diego, La Jolla, CA USA; 6 Department of Pediatrics/Rady Children’s Hospital San Diego, University of California San Diego, La Jolla, CA USA; 7 Beyster Center for Psychiatric Genomics, University of California San Diego, La Jolla, CA USA; 8 Kavli Institute for Brain and Mind, University of California San Diego, La Jolla, CA USA; 9 Center for Academic Research and Training in Anthropogeny (CARTA), La Jolla, CA USA

**Keywords:** Genetics, Neuroscience, Autism spectrum disorders

## Abstract

E3-ubiquitin ligase Cullin3 (*Cul3*) is a high confidence risk gene for autism spectrum disorder (ASD) and developmental delay (DD). To investigate how *Cul3* mutations impact brain development, we generated a haploinsufficient *Cul3* mouse model using CRISPR/Cas9 genome engineering. *Cul3* mutant mice exhibited social and cognitive deficits and hyperactive behavior. Brain MRI found decreased volume of cortical regions and changes in many other brain regions of *Cul3* mutant mice starting from early postnatal development. Spatiotemporal transcriptomic and proteomic profiling of embryonic, early postnatal and adult brain implicated neurogenesis and cytoskeletal defects as key drivers of *Cul3* functional impact. Specifically, dendritic growth, filamentous actin puncta, and spontaneous network activity were reduced in *Cul3* mutant mice. Inhibition of small GTPase RhoA, a molecular substrate of Cul3 ligase, rescued dendrite length and network activity phenotypes. Our study identified defects in neuronal cytoskeleton and Rho signaling as the primary targets of *Cul3* mutation during brain development.

## Introduction

**Fig. 1:**
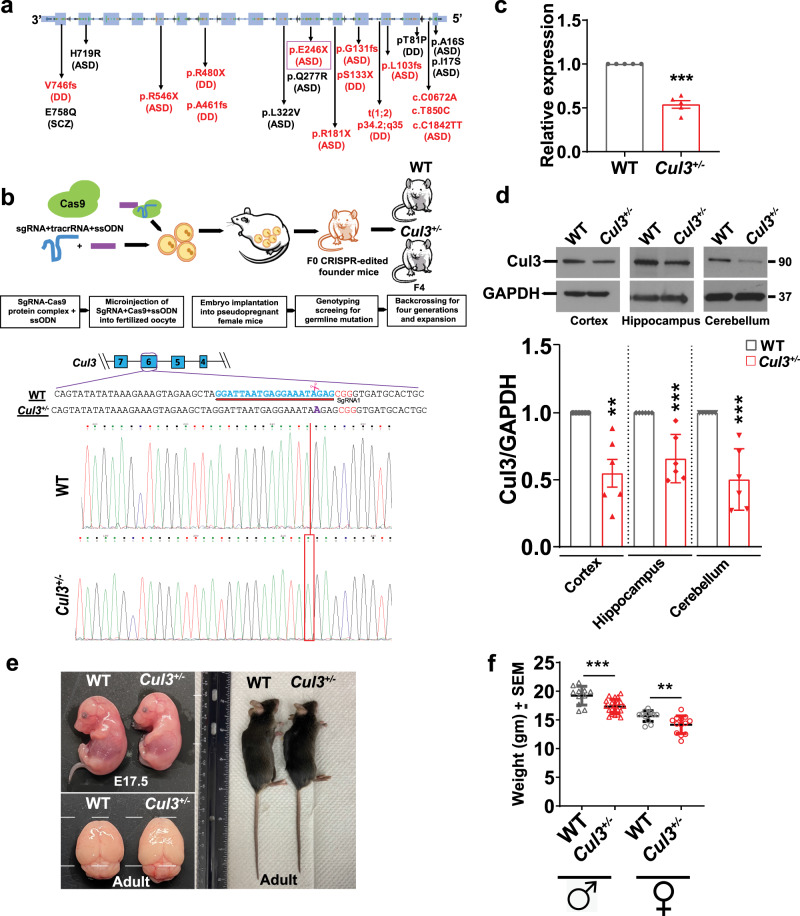
Generation and characterization of *Cul3*^+/−^ mouse model. **a** Loss-of-function (red font) and missense (black font) mutations in *Cul3* gene identified in the patients with neurodevelopmental disorders. ASD autism spectrum disorder, SCZ schizophrenia, DD developmental delay. The location of E246X patient’s mutation from this study is highlighted in the magenta-colored box. **b** Schematic representation of mouse line generation using CRISPR/Cas9 genome editing. cDNA sequence trace showing 1-bp insertion (purple) in exon 6 is shown. Single guide (Sg) RNA is shown in blue, PAM site is shown in red. Sanger sequencing diagram showing 1 bp insertion is at the bottom. **c** qRT-PCR showing reduction of mRNA expression of Cul3 in adult cerebral cortex of *Cul3*^+/−^ mice (***p < 0.001; two-tailed *t*-test; *n* = 5 for both genotypes); Error bars represents mean ± SEM. **d** Western blot of Cul3 protein, showing its significant reduction in *Cul3*^+/−^ mutant mice cortex (*p* < 0.01; *n* = 6 for each genotype); hippocampus (*p* < 0.001; *n* = 6 for each genotype) and cerebellum (*p* < 0.001; *n* = 6 for each genotype); error bars represents mean ± SD. **e** (Top left panel) Representative image of embryonic day E17.5 WT and *Cul3*^+/−^ embryo demonstrating smaller size; (bottom left panel) WT and *Cul3*^+/−^ adult brain; (right panel) representative image of adult (8-weeks old) WT and *Cul3*^+/−^ males demonstrating smaller body size of *Cul3*^+/−^ mutants. **f** Both adult male and female *Cul3*^+/−^ mice have reduced body weight as compared to their WT littermates (***p < 0.001; **p < 0.01; two-tailed *t*-test) (WT *n* = 11 male/12 female; *Cul3*^+/−^ *n* = 21 male/14 female); ○-female and ∆-male; Error bars represents mean ± SEM; two-tailed *t*-test was used for calculating statistical significance for (**c**–**f**). Dots represent individual animals.

Rare and de novo single nucleotide variants (SNVs) and copy number variants (CNVs) are major risk factors for neurodevelopmental disorders (NDDs). E3 ubiquitin ligase Cullin 3 (*Cul3*) is among the genes most confidently implicated in NDDs with genome-wide significance (FDR < 0.01) [1]. Exome sequencing studies have identified at least 20 mutations in the *Cul3* gene (13 protein-truncating mutations and 7 missense) in the patients with autism spectrum disorder (ASD), developmental delay (DD), and schizophrenia (SCZ), with no *Cul3* mutations detected in healthy controls (Fig. 1a, Supplementary Table S1). Other protein ubiquitination, deubiquitination, and degradation pathway genes (*Ube3A, Otud7A, Usp45, Cul1, Usp9x, Ubr1*, and many others) [1–5] have been shown to carry excess of mutations in patients, strongly implicating this pathway in NDDs.

Cul3 belongs to a family of RING E3 ubiquitin ligases, and its structure and function has been well-characterized, mostly in non-neuronal cell types [6]. Cul3 regulates turnover of a variety of substrate proteins by ubiquitinating and directing them for proteasomal degradation. Cul3 can interact with different adapter proteins, and is involved in regulation of a wide range of cellular processes, including cytoskeleton organization, cell fate determination, cell cycle regulation, and stress response among many others [6]. Less is known about the function of Cul3 in neural cells, despite its increased expression during embryonic stages of brain development [7]. Notably, homozygous deletion of *Cul3* in mice is embryonically lethal suggesting its crucial role during early development [8].

Interestingly, one of Cul3 adapter proteins, KCTD13, is located within a large 16p11.2 CNV that confers high risk for neurodevelopmental disorders [9]. We previously demonstrated that *Cul3* is co-expressed with *KCTD13*, and corresponding proteins physically interact during late mid-fetal period of brain development [10]. This interaction is crucial for regulating the levels of a small GTPase RhoA that controls actin cytoskeleton structure and cell movement [11]. Upregulation of RhoA through its constitutive expression has been linked to suppression of dendritic spine morphogenesis and to dramatic loss of spines [12, 13]. The contribution of RhoA to local regulation of axon growth has also been recently demonstrated [14].

To investigate how *Cul3* mutations impact early brain development at the molecular level, we generated *Cul3* haploinsufficient (*Cul3*^+/−^) mouse model using CRISPR/Cas9 genome engineering. We introduced a germline 1 bp insertion into exon 6 of *Cul3*, immediately adjacent to G754T (E246X) mutation detected in an ASD patient [15] (Fig. 1b). We extensively characterized *Cul3*^+/−^ mice at multiple levels, starting from brain anatomy and ending with transcriptomic and proteomic profiling of various brain regions at three developmental time points. Our results demonstrate that *Cul3* haploinsufficiency severely impacts early brain development through dysregulation of neuronal actin and intermediate filament cytoskeleton. Reduced dendrite growth and network activity of cortical neurons were among the most notable neuronal phenotypes observed in *Cul3*^+/−^ mice. Importantly, upregulation of one of the Cul3 substrates, small GTPase RhoA, a regulator of cytoskeletal dynamics, neuronal growth, and migration, linked observed molecular defects with Cul3 pathology. Treatment of cortical neurons with RhoA inhibitor Rhosin rescued dendritic length and neural network activity phenotypes.

## Results

### *Cul3*^+/−^ mice have brain anatomical defects starting from early postnatal period

To investigate the impact of *Cul3* mutations on brain development at the molecular level, we generated *Cul3*^+/−^ haploinsufficient mouse model on C57BL/6N background by introducing a 1 bp frame-shifting insertion into exon-6 of *Cul3* one base pair upstream from the nucleotide mutated in an ASD patient [15] using CRISPR/Cas9 genome editing (Materials and methods). We validated the presence of mutation by Sanger sequencing (Fig. 1b). In agreement with previous observations from conditional *Cul3*^*flox*^ mice [8], homozygous *Cul3*^*−/−*^ mutant animals were not viable. A heterozygous *Cul3*^+/−^ founder mouse harboring the insertion was expanded via breeding with wild-type (WT) C57BL/6N mice for at least four generations to eliminate possible CRISPR off-target effects. We verified reduced levels of *Cul3* transcripts in F_4_ *Cul3*^+/−^ mice by qRT-PCR (Fig. 1c), and reduced Cul3 protein levels by western blot (Fig. 1d). These experiments demonstrated the reduction of Cul3 expression in the brain of *Cul3*^+/−^ mutant mice to approximately half of the level observed in WT littermates, thereby validating *Cul3* haploinsufficiency.

The heterozygous *Cul3*^+/−^ mice were viable, reached a normal lifespan, and were fertile irrespective of sex. However, *Cul3*^+/−^ embryos (at both E15.5 and E17.5) and adult mice were smaller (Fig. 1e, Supplementary Fig. S1a). Adult mutant mice had slightly visually elongated brain and significantly reduced body weight (*P* < 0.001 for male and *P* < 0.01 for female; two-tailed *t*-test, Fig. 1f).

**Fig. 2:**
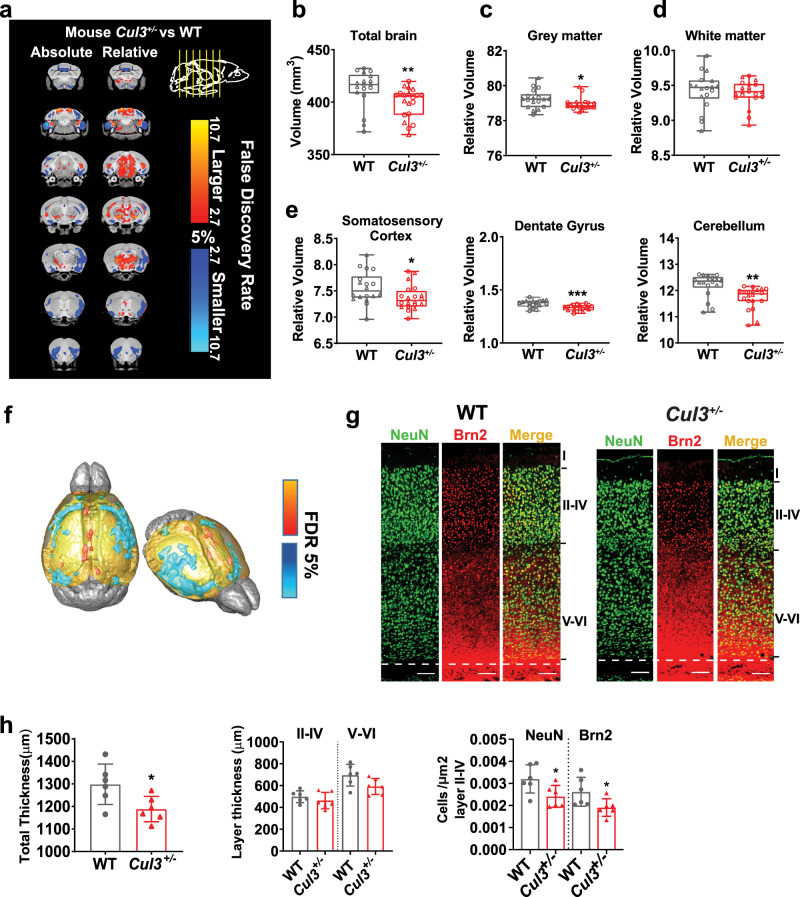
*Cul3*^+/−^ mice have altered brain morphology. **a** Voxel-wise analysis highlighting significant differences in relative volumes throughout the brain between WT and *Cul3*^+/−^ mice with 5% false discovery rate (FDR). Left panel is absolute changes and right panel is relative change in brain regions. Scale bar 2.7–10.7 indicates decreasing FDR, where 2.7 = 5% FDR. Red color signifies increased and blue color signifies decreased brain volume compared with WT brain. **b** *Cul3*^+/−^ mice have reduced absolute brain volume (**p < 0.01) compared to WT mice (WT *n* = 18, *Cul3*^+/−^ *n* = 20). **c** Reduced relative grey matter volume in *Cul3*^+/−^ mouse brain (*p < 0.05). **d** No significance difference is observed in relative white matter volume. **e** MRI revealed significant reduction in relative volume, normalized by total brain volume, of primary somatosensory cortex (*p < 0.05); hippocampal DG region (***p < 0.001); cerebellum (**p < 0.01). Dots represent individual animals; ○-female and ∆-male**. f** Reduced cortical thickness is observed by MRI in *Cul3*^+/−^ mice, turquoise color is decreased, red/orange color is increased. **g** Reduced cortical thickness of somatosensory cortex in adult *Cul3*^+/−^ mice, left panel shows somatosensory cortex region stained with NeuN (mature neuron marker) and BRN2 (layer II–IV marker); Scale bar is 100 μm. **h** Reduction of total cortical thickness, layer thickness and density of NeuN- and BRN2-positive cells/area in layer II–IV are observed (*p < 0.05; two-tailed *t*-test, *n* = 6 for each genotype). Dots represent independent samples; two-tailed *t*-test used for (**b**–**h**); error bars represent mean ± SD.

To investigate in more detail whether Cul3 mutation impacts brain neuroanatomy, we analyzed PFA-fixed 8- to 10-week-old adult WT and *Cul3*^+/−^ mouse brains (*N* = 38) using magnetic resonance imaging (MRI) (Materials and methods). We observed profound brain volume abnormalities in 46% (83/182, FDR < 5%) of brain regions in *Cul3*^+/−^ adult mice (Fig. 2a, Supplementary Table S2). Total brain volume, as well as relative grey matter volume, were both significantly lower in *Cul3*^+/−^ mice compared to WT (*P* < 0.01 and *P* < 0.05, respectively, two-tailed *t*-test, Fig. 2b, c). No significant differences in the white matter volume were observed between *Cul3*^+/−^ and WT mice, with a trend for lower volume in *Cul3*^+/−^ mice (Fig. 2d). With respect to specific brain regions, several areas of somatosensory cortex, hippocampal dentate gyrus and cerebellum were significantly decreased in *Cul3*^+/−^ mice (Fig. 2e). Among other notable brain areas, many olfactory bulb regions, primary visual cortex, entorhinal cortex, and corpus callosum were also decreased in the mutant (Supplementary Table S2). Overall, the volume of many cortical regions was decreased, whereas the volume of some subcortical regions was increased in the adult mutant mice.

It has been recently demonstrated that the impact of some NDD mutations could be sex-specific [16, 17]. Here, we investigated sex-specific effect of Cul3 haploinsufficiency by analyzing brain MRI results for male and female mice separately. The adult *Cul3*^+/−^ males had greater number of altered brain regions (62/182) than females (44/182) at 10% FDR (Supplementary Fig. S2). Relative volumes of some brain regions (Hippocampal CA1, CA2, DG regions, fornix, basal forebrain, nucleus accumbens) were altered more drastically in adult males, whereas claustrum, insular region, cingulate cortex and ectorhinal cortex were more affected in females (Supplementary Table S2; Supplementary Fig. S2). Overall, the majority of brain regions followed similar up- or downward trends in both sexes. This implies that Cul3 mutation impacts both sexes in a similar way, but the adult *Cul3*^+/−^ males have relatively greater number of altered regions compared to females.

To understand whether brain changes in the mutant mice initiate early in development, we also performed MRI on fixed WT and *Cul3*^+/−^ mouse brains (*N* = 41) at postnatal day 7 (P7) (Supplementary Fig. S1, Supplementary Table S2). Similar trends in brain volume changes were found in the early postnatal P7 period as in the adult, with significant decrease in total brain volume, frontal cortex, parieto-temporal cortex, and cerebellum in the mutant mice. Hypothalamus was increased in P7 mice. Interestingly, sex-specific effect of Cul3 mutation at P7 was stronger in females than in males. The females had greater number (19/56) of altered brain regions than males (0/56) at 10% FDR (Supplementary Fig. S2, Supplementary Table S2). These results suggest that the impact of *Cul3* mutation initiates during early brain development, but it is more pronounced in the adulthood.

The MRI data suggests that besides volumetric changes, *Cul3*^+/−^ mice displayed decreased cortical thickness, particularly within frontal cortical regions of the brain (Fig. 2f). To investigate what layers contributed to the observed reduced thickness, we immunostained adult somatosensory cortex with mature neuron marker NeuN and upper layer neuron (II–IV) marker BRN2 (Fig. 2g). We observed significant overall reduction of cortical thickness in mutant mice (*P* < 0.05, two-tailed *t*-test, Fig. 2h, left panel), even though the thickness of individual layers (II–IV and V–VI) demonstrated the trend but did not reach statistical significance. In addition, we observed reduction in NeuN- and BRN2-positive neuron number in layers II–IV in the mutant mice (*P* < 0.05, two-tailed *t*-test, Fig. 2h, right panel). This reduction in neuron number is likely due to premature neuronal death, as evidenced by the increased apoptosis measured by terminal deoxynucleotidyl transferase dUTP nick end labeling (TUNEL) in the primary cortical neurons at DIV14 (Supplementary Fig. S3). In summary, Cul3 mutation impacts overall embryonic development along with brain volume, cortical thickness, and neuron number starting from early postnatal periods.

### *Cul3* mutation impacts the behavior of *Cul3*^+/−^ mice

**Fig. 3:**
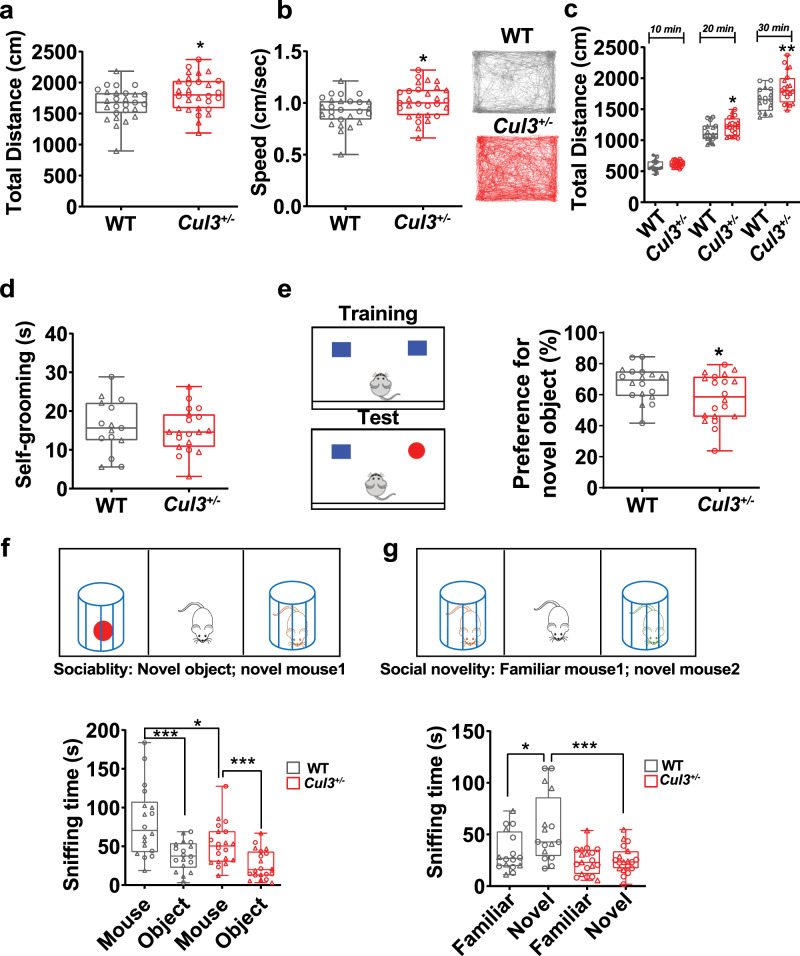
*Cul3*^+/−^ mice display hyperactivity, cognitive, and social impairments. **a** *Cul3*^+/−^ mice travel longer distances in open field (*n* = 27 WT; *n* = 29 *Cul3*^+/−^ (*p < 0.05)) compared with WT mice. **b** The traveling speed (cm/s) is significantly increased in *Cul3*^+/−^ mice (*p < 0.05); representative traces of 30 min in open field show *Cul3*^+/−^ mice traveling longer distances compared to WT mice. **c** Time bins showing that *Cul3*^+/−^ mice travel significantly longer distance in the last 20 min of the test (*p < 0.05; **p < 0.01). **d** No difference in time spent self-grooming between WT and *Cul3*^+/−^ mice (WT *n* = 15; *Cul3*^+/−^ *n* = 19). **e** *Cul3*^+/−^ mice demonstrate significantly reduced preference for novel object in novel object recognition test (*p < 0.05; two-tailed *t*-test, WT *n* = 18; *Cul3*^+/−^ *n* = 20). **f** (Upper panel) Schematic diagram of three-chamber social interaction test; reduced sniffing time is observed for *Cul3*^+/−^ mice while interacting with a novel mouse (vs novel object) as compared to WT mice (WT *n* = 17; *Cul3*^+/−^ *n* = 19; *p < 0.05, One-way ANOVA). **g** Reduced sniffing time is observed for *Cul3*^+/−^ mice while interacting with a novel mouse (vs familiar mouse) as compared to WT mice (WT *n* = 17; *Cul3*^+/−^ *n* = 19); ***p < 0.01; One-way ANOVA. Dots represent individual animals; ○-female and ∆-male; error bars represent mean ± SD.

Although the main goal of this study was to identify molecular pathways disrupted by Cul3 mutation, we performed limited behavior testing of *Cul3*^+/−^ animals for hyperactivity and social interaction. We would like to emphasize that complete behavior characterization of this mouse model was not the goal of this study. In the open field test, *Cul3*^+/−^ mice traveled longer distances (*P* = 0.032; two-tailed *t*-test, Fig. 3a) with higher speed (*P* = 0.032; *t*-test; Fig. 3b), suggesting hyperactivity. Data analysis in 10 min time bins revealed that hyperactivity of mutant animals increased after 10 min from the beginning of the test (Fig. 3c). To assess whether hyperactivity is related to anxiety, we measured time spent in the center of the open field arena vs periphery, and observed no difference between WT and *Cul3*^+/−^ animals (Supplementary Fig. S4). Self-grooming that may also suggest anxiety was not increased in the mutant mice (Fig. 3d). These results suggest that *Cul3*^+/−^ mice are hyperactive but not anxious.

Autism spectrum disorder is characterized by impairment in multiple domains, including memory and social functioning. To investigate whether Cul3 mutation impacts learning, short-term memory, and social interaction, we used novel object recognition test. In this task, *Cul3*^+/−^ mice spent shorter time exploring novel object than WT mice (*P* < 0.05, two-tailed *t*-test, Fig. 3e). We then performed a three-chamber social interaction test, in which we first measured the sociability by analyzing the sniffing time of novel mouse vs novel object. Both *Cul3*^+/−^ and WT mice preferred the mouse to the object, but *Cul3*^+/−^ mice spent slightly less time sniffing the mouse than WT mice (*P* < 0.001, One-way ANOVA, Fig. 3f). However, when a novel mouse was introduced instead of the object in the social novelty phase, *Cul3*^+/−^ mice spent the same time sniffing novel and familiar mouse, and this sniffing time was shorter in mutant compared to WT mice (*P* < 0.001, One-way ANOVA, Fig. 3g). These results suggest that *Cul3*^+/−^ mice have impaired short-term memory and social interaction.

The sex-specific analyses of behavior demonstrated that both, male and female *Cul3*^+/−^ mice, had significant similarities. However, we did observe sex-specific effect in the open field test. In this test, the total distance (*P* < 0.05, two-tailed *t*-test) as well as distance traveled in 10 min bins (*P* < 0.01; *P* < 0.05, two-tailed *t*-test) were significantly higher for *Cul3*^+/−^ males, but not for *Cul3*^+/−^ females (Supplementary Fig. S4). The *Cul3*^+/−^ males, but not *Cul3*^+/−^ females, also demonstrated significantly higher speed in the open field test. This result emphasizes that males are relatively more hyperactive compared to *Cul3*^+/−^ females. Interestingly, in the social novelty phase of the three-chamber social interaction test, we observed that both *Cul3*^+/−^ males and females have significant reduction in sniffing time for novel mouse compared to familiar mouse. This suggests both *Cul3*^+/−^ males and females have reduced sociability compared to their WT littermates (Supplementary Fig. S4). Self-grooming and novel object preference did not show sex-specific differences. Overall, Cul3 haploinsufficiency has robust effect on both sexes.

Since we observed changes in brain anatomy starting from early postnatal period, we investigated general developmental milestones in newborn mice through 21 days of age, in addition to behavioral testing of adult mice. There were no differences between genotypes in the eye opening, ear-twitching, auditory startle, forelimbs grasping, and other developmental milestones (Supplementary Fig. S5). The *Cul3*^+/−^ mice demonstrated delayed performance in surface righting and cliff aversion tests, suggesting potential problems with motor control and vestibular difficulties. However, motor control deficiency did not persist into adulthood as demonstrated by longer distance traveled and higher speed in the open field test for adult mutant mice (Fig. 3a–c).

### *Cul3* mutation dysregulates neurogenesis and cytoskeletal genes transcriptome-wide

Prior to investigating the impact of *Cul3* mutation on the developing mouse brain, we first analyzed *Cul3* expression in publicly available human and mouse brain transcriptome datasets. According to the BrainSpan transcriptome of the developing human brain [7], *Cul3* gene is highly expressed in neocortex during early human embryonic development (8pcw-13pcw), and its expression gradually decreases in late fetal, and especially in postnatal periods (Supplementary Fig. S6). In mice, *Cul3* expression was also higher in embryonic periods than in postnatal or adult, suggesting that the *Cul3* gene may play an important role in early brain development in both species. Single cell RNA sequencing of the human and mouse brain [18–20] demonstrated that *Cul3* is expressed at higher levels in neurons compared to other cell types in both species, with second highest expression in microglia/microphage in humans, but not in mice. This suggests that disruption of *Cul3* expression may have a greater impact on neuronal cell types compared to other cell types, at least in the mouse.

Since *Cul3* expression is the highest at embryonic periods, and we have also observed abnormalities in early postnatal and adult brain, we performed bulk RNA sequencing (RNA-seq) of 108 brain transcriptomes derived from three developmental periods (embryonic E17.5, early postnatal P7, and adult 4–6 weeks) and three brain regions (cortex-CX, hippocampus-HIP, and cerebellum-CB) of *Cul3*^+/−^ mutant and WT mice (Supplementary Fig. S7). The goal of transcriptome profiling was to detect how *Cul3* mutation affects other genes at the transcriptional level. After rigorous quality control and normalization (Supplementary Fig. S8), we performed differential gene expression analyses to identify genes that were up- or downregulated in the *Cul3*^+/−^ mutant *vs* WT mice (Materials and methods).

**Fig. 4:**
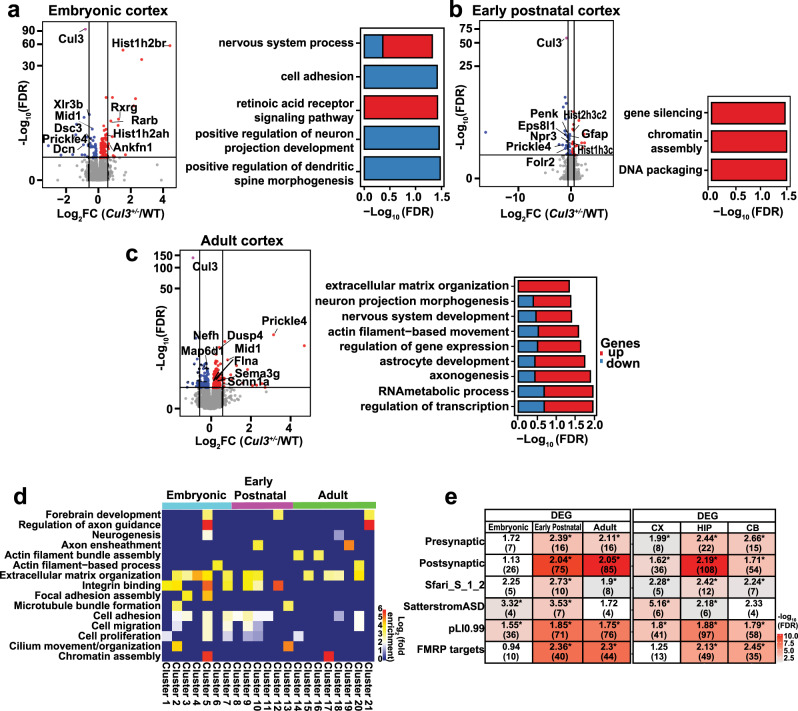
Spatiotemporal differential gene expression analyses identifies dysregulation of cytoskeletal processes by *Cul3* mutation. **a–c** Differential gene expression analyses of cortical samples from embryonic (**a**), early postnatal (**b**), and adult (**c**) developmental periods. (Left panels) Volcano plots of differentially expressed genes in *Cul3*^+/−^ vs WT. Genes colored in red are upregulated in *Cul3*^+/−^ compared to WT; genes colored in blue are downregulated in *Cul3*^+/−^ compared to WT; Cul3 is colored in pink. (Right panels) GO-terms enrichment of differentially expressed genes. Contribution of up- or downregulated genes to specific GO terms are shown in blue and red, respectively. **d ** Heatmap of enriched common GO terms at different developmental time periods. Fisher *P*-value combination analysis (Materials and methods) was applied to identify 21 clusters of differentially expressed up- and downregulated genes impacted by Cul3 mutation across developmental periods. Biological processes impacted in two or more clusters are shown (Individual cluster details are present in Supplementary Table S6). **e ** Enrichment of differentially expressed genes from each period and region with literature-curated gene lists with previous evidence for involvement in autism. These lists include pre- and post-synaptic genes from SynaptomeDB; syndromic and highly ranked (1 and 2) genes from SFARI Gene database (https://gene.sfari.org/database/gene-scoring/); genes with probability of loss-of-function intolerance (pLI) > 0.99 as reported by the Exome Aggregation Consortium; constrained genes; and FMRP target genes. Number of overlapped genes (in parenthesis) and odds ratio are indicated inside each cell, and provided only for FDR < 0.05 and OR > 1.

As expected, *Cul3* was the most differentially expressed protein-coding gene in all datasets, validating haploinsufficiency **(**Fig. 4a–c and Supplementary Fig. S9). Dosage changes of the *Cul3* gene also had transcriptome-wide effect, dysregulating other genes. We identified hundreds of differentially expressed genes (DEG) between *Cul3*^+/−^ mutant and WT mice at 10% FDR across developmental periods and brain regions (Supplementary Table S3). We identified 736, 1239, and 1350 unique DEGs in embryonic, early postnatal, and adult periods (by combining brain regions), respectively. Likewise, we identified 727, 1641, and 1032 unique DEGs in CX, CB, and HIP (by combining developmental periods), respectively. Higher number of shared DEGs was observed between embryonic vs early postnatal (*N* = 226) and early postnatal *vs* adult (*N* = 255) periods, with the lowest number in embryonic *vs* adult (*N* = 156) (Supplementary Fig. S9). Likewise, a higher number of shared DEGs was observed between CB vs HIP (*N* = 362), followed by CX vs CB (*N* = 182) and CX vs HIP (*N* = 159). gene ontology (GO) functional annotations of DEGs revealed enrichment of GO terms shared among multiple periods and regions as well as those that are unique to specific periods and regions (Supplementary Table S4). For example, GO functions of DEGs in CX included mostly neuronal processes (neuron projection, dendritic spine morphogenesis, axonogenesis), whereas HIP DEGs were enriched in cilium and cell adhesion. On the other hand, the DEGs in CB were enriched in synaptic and ion transport GO functions.

To determine whether *Cul3* mutation has sex-biased transcriptomic impact, we performed DEG analyses for males and females separately using the same model that was applied for the sex-blind analyses, where sex was used as a confounder. We identified 165/157, 170/253, and 303/106 DEGs in embryonic, early postnatal, and adult cortex in males/females, respectively; 68/429, 588/646, and 54/292 in embryonic, early postnatal and adult cerebellum in males/females, respectively; and 1684/215, 322/749, and 87/195 in embryonic, early postnatal and adult hippocampus in males/females, respectively. GO analyses of these sex-specific DEGs with 10% FDR were compared between males and females, and also to the GO terms from our previous sex-blind analyses. Most GO terms identified by the sex-specific analyses overlapped with those from the sex-blind analyses, however, we also found some GO terms that were exclusively sex-specific. For example, in embryonic cortex, nervous system process was shared by males and females, but two other GO terms (GABA-A receptor activity and notochord development) were male-specific, whereas cell migration and synaptic signaling were female-specific. Similarly, synaptic GO functions were female-biased in the P7 cortex, but were shared by both genders in the adult cortex. The lists of sex-specific GO terms in each brain region for each developmental period are shown in Supplementary Fig. S10 and the Supplementary Table S5. Overall, although we find sex-specific GO terms across different brain regions/periods, they for the most part recapitulate the terms that we have detected in our sex-blind analyses.

To better understand how gene expression is disrupted by the *Cul3* mutation across all periods and regions, we next carried out meta-analyses of DEGs (Materials and methods). This combined analysis identified clusters of genes that were either up- or downregulated in all periods or regions (Supplementary Table S6). GO annotation of these clusters highlighted terms shared by at least two clusters (Fig. 4d and Supplementary Table S7). Biological processes, such as neurogenesis and axon guidance, integrin and extracellular matrix, cell adhesion, migration, and proliferation, as well as actin cytoskeleton were altered in multiple clusters suggesting that Cul3 affects these processes transcriptome-wide.

To put our DEGs into a context of the existing knowledge of autism genetics, we performed statistical enrichment analyses of DEGs against curated gene lists with previous evidence for involvement in ASD (Fig. 4e). We observed enrichment of embryonic and early postnatal DEGs in high confident ASD risk genes from Satterstrom (OR > 3) [5]. Early postnatal and adult DEGs were enriched in presynaptic, postsynaptic, and FMRP binding targets. DEGs from all three periods were enriched in genes highly intolerant to mutations (pLI > 0.99). With regards to regions, CX was enriched in ASD risk genes from Satterstrom et al. (OR > 5), and all three brain regions were enriched in presynaptic and postsynaptic, ASD risk genes from SFARI, and genes highly intolerant to mutations (pLI > 0.99). This suggests that *Cul3* mutation dysregulates genes relevant to ASD pathogenesis, especially in the cortex and during embryonic and early postnatal periods. This is consistent with human data highlighting the impact of ASD mutations during late mid-fetal cortical development [21, 22].

### Proteomic profiling supports neuron cytoskeleton and neuron projection development dysregulation

Cul3 is a part of the ubiquitin-proteasome system that has a significant role in protein turnover. Cul3 ubiquitin ligase interacts with a number of adapter proteins to ubiquitinate various substrates and to direct them for proteasomal degradation. Given the role of Cul3 in posttranscriptional regulation, we expect that *Cul3* mutations could impact a cell’s proteome to a greater degree than they impact the transcriptome. To investigate the impact of Cul3 on brain proteome, we carried out quantitative Tandem Mass Tag mass spectrometry (TMT-MS) on 48 brain samples derived from three developmental periods (embryonic E17.5, early postnatal P7 and adult 4–6 weeks) and two brain regions (cortex CX and cerebellum CB) of *Cul3*^+/−^ mutant and WT mice (Supplementary Fig. S11). Differential protein expression analyses identified hundreds to thousands of differentially expressed proteins (DEPs) across various datasets (Supplementary Table S8). Overall, a greater number of DEPs was detected in embryonic brain vs early postnatal or adult, and in CX vs CB (Supplementary Fig. S12).

**Fig. 5:**
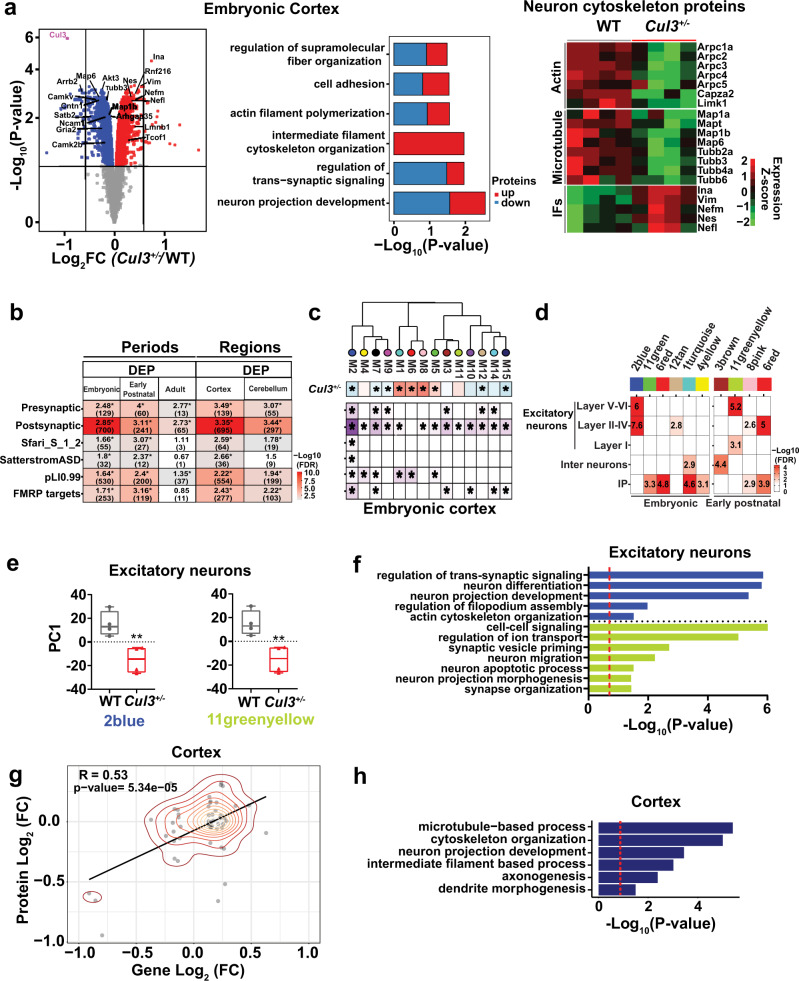
Differential protein expression and weighted protein co-expression network analyses of *Cul3*^+/−^ mice. **a** (Left panel) Volcano plot of differentially expressed proteins between *Cul3*^+/−^ and WT embryonic cortex identified from quantitative TMT-MS proteomic profiling. Cul3 is colored in pink, proteins colored in red are upregulated and proteins colored in blue are downregulated in *Cul3*^+/−^embryonic cortex. (Middle panel) Gene ontology enrichment analyses of up- and downregulated proteins are shown as bar plots. Contribution of up- or downregulated proteins to specific GO terms are shown in blue and red, respectively. (Right panel) Expression Heatmap of proteins associated with actin, microtubule, and intermediate filament cytoskeleton in WT and *Cul3*^+/−^ embryonic cortex. **b** Enrichment of differentially expressed proteins (combined by period and region) with literature-curated gene lists with previous evidence for involvement in autism. Number of overlapped proteins (in parenthesis) and odds ratio are shown. **c** Hierarchical clustering of protein co-expression modules by module eigengene for *Cul3*^+/−^ embryonic cortex. Module-genotype associations (* FDR < 0.1) for each module are shown below dendrogram. A total of 9 modules were significantly associated with *Cul3*^+/−^ genotype in embryonic cortex. Module enrichment analyses against literature-curated gene lists with previous evidence for involvement in autism are shown at the bottom (* FDR < 0.05) inside each cell, and provided only for FDR < 0.05 and OR > 1. Row annotations are the same as in panel (**b**) starting from presynaptic proteins in row one and ending with FMRP targets in row six. **d** Cell type enrichment of co-expression modules from embryonic cortex using P0 mouse cortex scRNA-seq dataset [24]. Modules significantly enriched in at least one cell type are shown for embryonic and early postnatal time periods. **e** PC1 of representative modules with significant cell type enrichment plotted by genotype. Embryonic module *2blue* and early postnatal module *11greenyellow* from *Cul3*^+/−^ are depleted in excitatory neurons. All comparisons between WT and *Cul3*^+/−^ are significant using *t*-test statistics. **f** GO terms for *2blue* and *11greenyellow* modules were obtained using g:Profiler [57]. **g** Transcriptome vs proteome correlation for cortex. DEGs for cortex from all developmental periods were overlapped with proteomes to extract corresponding proteins.  The Pearson’s correlation coefficient for DEGs and corresponding proteins expression fold changes from cortex is shown, each dot is one gene/protein. **h** GO term enrichment analysis of correlated genes/proteins for cortex, highlighting cytoskeletal and neurogenesis functions.

GO annotations pointed to dysregulation of neuron projection, synaptic signaling, and cytoskeletal functions (Fig. 5a). The neuron projection, intermediate filament, and ion transport GO functions were shared among different periods (Supplementary Table S9). Notably, three cytoskeletal proteins, plastin 3 (Pls3), and neuronal intermediate filament proteins internexin (Ina) and vimentin (Vim), were found to be upregulated in all proteomics datasets, supporting neuron cytoskeleton dysregulation by the *Cul3* mutation with higher confidence (Fig. 5b). We confirmed Pls3 upregulation by western blot (Supplementary Fig. S12).

Among differentially expressed cytoskeletal proteins, only intermediate neurofilament proteins (Vim, Nes, Ina, Nefl, Nefm) were significantly upregulated, whereas neuron microtubule or actin filaments proteins (Tubb2, Tubb3, Map1b, Actn1, Actn4, Limk, Arpc3, Arpc4, Capza2) were downregulated in the mutant embryonic mouse cortex (Fig. 5a). The fine balance of intermediate neurofilaments, microtubules, and actin cytoskeleton is required for early cell positioning, polarization and neuritogenesis [23]. Dysregulation of these proteins suggests potential impact on neuron growth and development during early embryonic development of *Cul3*^+/−^ cortex. Interestingly, small GTPase RhoA, one of the substrate protein for Cul3 and regulator of actin cytoskeleton and neurite growth, was also upregulated in embryonic cortex and cerebellum. In addition, several other proteins with important neuronal and developmental functions were shared among datasets. They include receptor for netrin Dcc, required for axon guidance, contactin-associated protein-like Cntnap2, and treacle ribosome biogenesis factor Tcof1, involved in embryonic development of the craniofacial complex. Similar to DEGs, we performed enrichment analyses of DEPs with genes previously implicated in brain development and ASD. Embryonic and early postnatal developmental periods demonstrated stronger enrichment of genes from essentially all tested datasets, including ASD risk genes (Fig. 5b). Likewise, CX had stronger enrichments across all datasets than CB. This suggests that Cul3 mutation may have greater impact on proteins involved in early cortical growth and development.

Besides differentially expressed proteins, the modules of proteins with correlated expression (i.e., co-expression modules) could provide further insight into pathways disrupted by Cul3 mutation. The weighted protein co-expression network analysis (WPCNA) identified multiple protein co-expression modules positively or negatively associated with *Cul3*^+/−^ genotype (Supplementary Table S10). We detected twenty-one co-expression modules in CX (9 embryonic, 5 early postnatal and 7 adult) and eleven modules in CB (8 embryonic, 1 early postnatal and 2 adult) that were positively or negatively associated with *Cul3*^+/−^ genotype (Supplementary Table S10). GO annotations of CX (Supplementary Table S11) and CB modules (Supplementary Table S12) were in agreement with biological functions identified from DEG and DEP analyses. For example, *8 pink* module was upregulated in *Cul3*^+/−^ embryonic CX and enriched in intermediate filament proteins (Vim, Ina, Nes). Likewise, downregulated *2blue* module was enriched in neuronal and synaptic functions (neuron projection development, neuron differentiation, regulation of trans-synaptic signaling), with many ASD risk genes found within this module (CTNNB1, DPYSL2, NRXN1, STX1B, TBR1). Several modules contained proteins associated with chromatin and histone modification functions (*1turquoise* and *6red* in embryonic CX, and *6red* in early postnatal CX), and these modules were all upregulated in the *Cul3*^+/−^ mouse. Further analyses demonstrated significant enrichment of almost all modules in postsynaptic proteins (Supplementary Fig. S13), whereas downregulated *2blue* module in embryonic CX was enriched in ASD risk genes from Satterstrom [5] (Fig. 5c). In summary, the WPCNA analyses of Cul3 spatio-temporal brain proteome further confirm dysregulation of neuronal and cytoskeletal protein modules by Cul3 mutation.

To better understand what cell types are impacted by the *Cul3* mutation, we performed enrichment analyses of our CX modules with scRNA-seq data from the P0 developing mouse neocortex [24]. We observed several modules enriched in various cell types, with strongest enrichment of excitatory neurons from cortical layers II–VI in embryonic CX *2blue* and early postnatal CX *11green-yellow* modules (Fig. 5d). Both modules were downregulated in *Cul3*^+/−^ mice (Fig. 5e), and their GO functions consisted on neuronal and synaptic functions such as “neuron projection development”, “neuron differentiation” and “trans-synaptic signaling” (Fig. 5f). These results suggest that Cul3 mutation may impact excitatory neurons in the developing cortex of *Cul3*^+/−^ mice.

We next asked whether transcriptomic and proteomic analyses point to shared pathways dysregulated by the *Cul3* mutation. To address this question, we performed transcriptome vs proteome correlation analysis for cortex and cerebellum by overlapping DEGs that we have identified with the entire proteomes for each brain region. We computed Pearson correlation coefficient (*R*) and *p*-values between log_2_ (FC) of genes vs proteins for cortex and cerebellum individually for each period, and also by combining all developmental periods for each region to increase power. The individual *R*-values ranged from 0.35 to 0.59 for the cortex and from 0.23 to 0.4 for the cerebellum across periods (Supplementary Table S13). The *R* values for combined cortex (*R* = 0.53) and cerebellum (*R* = 0.22) across all periods were in the positive range, with higher correlation observed for the cortex (Fig. 5g). The GO terms spanned neuronal and cytoskeletal functions, axonogenesis and dendrite morphogenesis for cortex, and cytoskeletal and synaptic functions, glial cell development, and myelination for cerebellum (Supplementary Fig. S13). Overall, the GO functions from both, individual transcriptome and proteome analyses, as well as from the correlated combined analyses, converged on similar processes/pathways, thereby further strengthening our findings.

### *Cul3* haploinsufficiency leads to reduced dendritic length and decreased neuronal network activity in primary cortical neurons

The DEGs, DEPs and co-expression modules in our transcriptomic and proteomic analyses were consistently enriched in neurogenesis, neuron projection development, and synaptic GO functions. We observed up- or downregulation of a number of proteins involved in dendrite and axon growth (Map2b, Tubb3, EphA7, DPYSL2, RAB family proteins), as well as cytoskeletal proteins involved in regulation of actin polymerization from Arp2/3 and WASP complexes (ARPC2, ARPC3, EVL, BAIAP2). In addition, our data demonstrate that *Cul3* dysregulates early brain development, with potentially greater impact on cortical neuron growth and development.

To investigate the impact of *Cul3*^+/−^ on early cortical development and neurogenesis, we performed immunostaining on the fixed E14 WT and *Cul3*^+/−^ brain cortical sections. We observed a trend towards reduction of cortical thickness in E14 *Cul3*^+/−^ mice compared to WT (Supplementary Fig. S14), which is consistent with our previous findings from the adult mice (Fig. 2h). These data suggest that defects in cortical development in *Cul3*^+/−^ mice begin early on, starting from E14, or perhaps even earlier. Immunostaining with markers for specific cell type populations, such as Sox2 for progenitors, Ki67 for cell cycling, and TBR1 for post-mitotic neuronal cells in the developing cortex demonstrated no significant difference between WT and *Cul3*^+/−^ for Sox2-positive neural progenitor pool as well as for Ki67-positive proliferating cell population at E14. However, we observed significant reduction of the number of TBR1-positive neurons in *Cul3*^+/−^ (Supplementary Fig. S14). This finding is consistent with our previous observation of reduction in NeuN-positive neurons in the adult *Cul3*^+/−^ mouse cortex (Fig. 2h). Thus, decrease of TBR1-positive neurons in the embryonic brain and NeuN-positive neurons in the adult brain of *Cul3*^+/−^ mice, along with reduced cortical thickness in both developmental periods, collectively suggests potential defects in neuron differentiation/generation and/or migration that start early in development. Overall, the results from embryonic cortex recapitulate those observed in the adult mice, suggesting that defects in neurogenesis precede the changes observed in the adulthood.

**Fig. 6:**
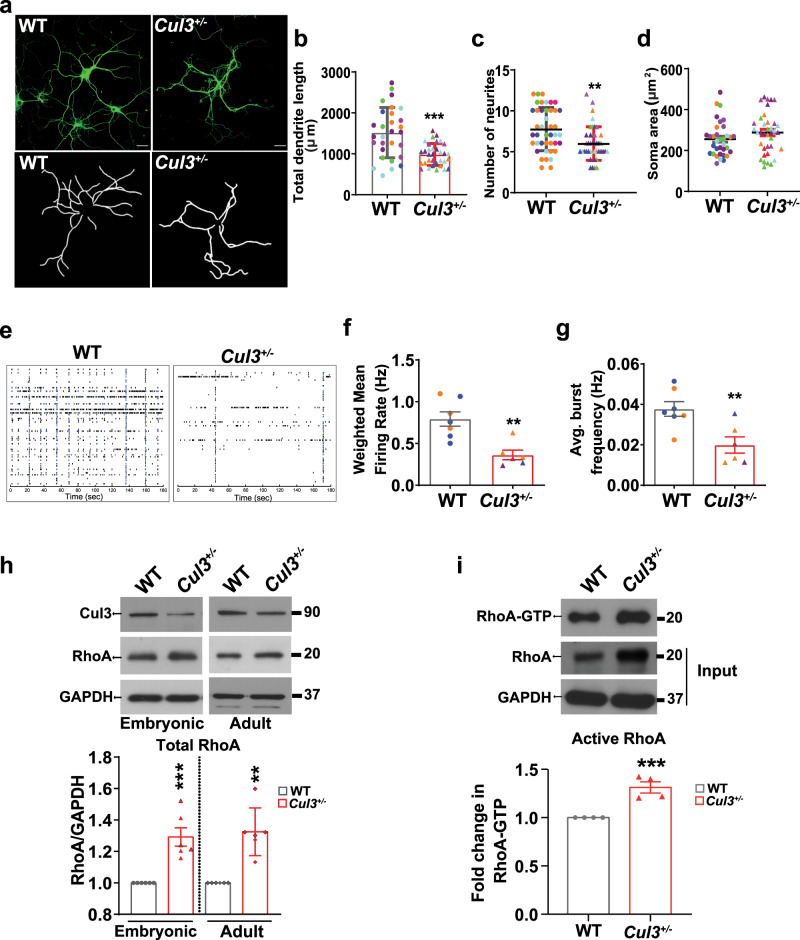
*Cul3* haploinsufficiency leads to altered neuron growth, network activity, and to RhoA upregulation in *Cul3*^+/−^ mice. **a** Representative images of 14DIV primary cortical neurons from WT and *Cul3*^+/−^ mice, immunostained with MAP2 (upper panel), and tracings by simple neurite tracer (lower panel). Scale bar is 25 µm. **b**–**d** Quantification of total dendrite length, neurite number, and soma size are shown. Symbols represent independent neurons and color represents littermates. Data are shown as mean ± SD (*n* = 6 per genotype, at least 6–8 neurons per mouse). Significance is calculated using two-tailed *t*-test; ***p < 0.001; **p < 0.01. **e** Representative raster plots of spontaneous spike activity from 8DIV primary cortical neurons. **f** Spontaneous spike activity is significantly reduced in *Cul3*^+/−^ cortical neurons; **p < 0.01, two-tailed *t*-test. **g** Average burst frequency is significantly reduced in *Cul3*^+/−^ neurons; **p < 0.01; two-tailed *t*-test, *n* = 6–7 mice per genotype; 1.5 × 10^6^ neurons were seeded from each mouse in each MEA plate well, each containing 64 electrodes. Each dot represents independent mouse and color represents littermates. **h** Representative images of western blot analysis of Cul3, total RhoA, and GAPDH loading control in embryonic and adult cortices. Densitometry analysis of western blot is shown at the bottom. Data are presented as mean ± SEM (*n* = 6 per genotype). **i** Representative images of western blot analysis of active RhoA (RhoA-GTP) pulldown, total RhoA and GAPDH loading control from input lysate of embryonic cortex. Densitometry analysis of western blot is shown at the bottom. Data are presented as mean ± SEM (*n* = 4 per genotype for total RhoA, *n* = 4 per genotype for active RhoA). Significance is calculated using two-tailed *t*-test; **p < 0.01. The significance above bars represents comparison against WT.

Further, to investigate morphological defects of neurons in *Cul3*^+/−^ mice, we examined neuron morphology of primary cortical neurons derived from E17.5 embryonic *Cul3*^+/−^ mutant and WT mice. We calculated total dendrite length and the number of neurites, along with soma size, of 14DIV neurons stained with Map2 dendritic marker (Fig. 6a). Tracing of Map2-positive dendrites demonstrated reduction of total dendritic length and the number of neurites in *Cul3*^+/−^ mice (*P* = 0.001; two-tailed *t*-test; Fig. 6b, c). Soma size was not affected by the *Cul3* mutation (Fig. 6d).

Altered neuron morphology, and especially decreased dendritic length, could impact synaptic connectivity and alter neuronal network activity. We next examined network activity using multielectrode array (MEA) recordings in 8 days old primary cortical neuronal culture. Representative raster plots of spontaneous spike activity from these cultures are shown in Fig. 6e. Analyses of spontaneous neural recordings demonstrated significantly reduced weighted mean firing rate in *Cul3*^+/−^ neurons compared to WT (*P* = 0.002, two-tailed *t*-test; Fig. 6f). *Cul3*^+/−^ neurons also had significantly reduced bursting activity (*P* = 0.007, two-tailed *t*-test; Fig. 6g). These results suggest neural network activity defects in the mutant mice.

We next sought to search for the molecular target involved in dendritic deficits and reduced network activity of *Cul3*^+/−^ cortical neurons. Based on our data, there are several indicators that point to potential dysregulation of Rho signaling in *Cul3*^+/−^ mouse brain. First, proteomic profiling identified a small GTPase RhoA as significantly upregulated protein in embryonic cortex and cerebellum. Second, RhoA is one of the direct molecular substrates of Cul3 ubiquitin ligase complex. Cul3 ubiquitinates RhoA and directs it for proteasomal degradation [11]. Third, RhoA is an important regulator of actin cytoskeleton, neuron growth, and migration during early brain development [12]. Since we observed defects in neuronal growth, neurogenesis and neuron cytoskeleton, we tested for potential involvement of RhoA by western blot in embryonic and adult CX. We observed significant upregulation of total RhoA in both tissues (*P* < 0.001 for embryonic and *P* < 0.01 for adult, two-tailed *t*-test, Fig. 6h). We also tested the level of GTP-bound active form of RhoA (RhoA-GTP) and observed its significant increase in embryonic CX, in agreement with total RhoA upregulation (*P* < 0.001, two-tailed *t*-test, Fig. 6i). Increased RhoA levels during development may lead to growth cone retraction and altered neurite growth and extension.

### *Cul3* mutation destabilizes actin cytoskeleton and leads to loss of F-actin puncta in cortical neurons

**Fig. 7:**
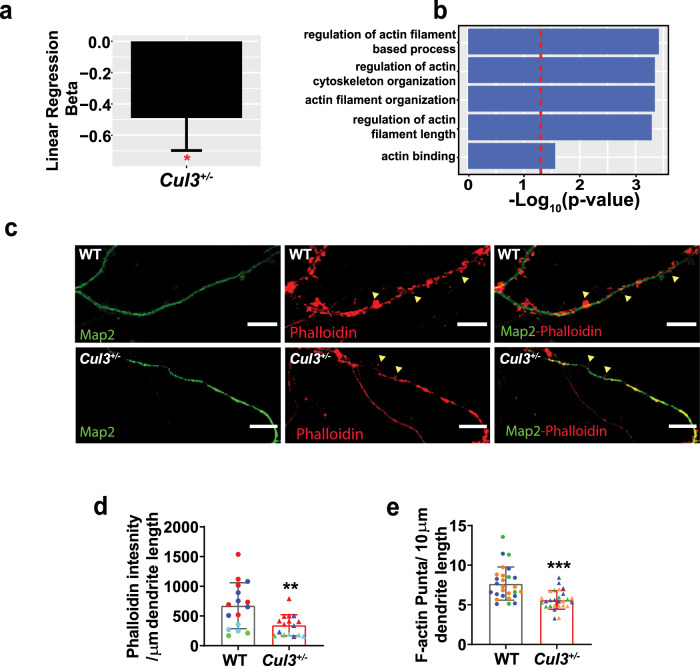
Actin cytoskeleton defects are observed in *Cul3*^+/−^ mice. **a**, **b** Module-genotype association and GO functional annotations for *black* actin module identified by protein co-expression analyses of adult cortex. (*n* = 4 per genotype). **c** Representative images of 21DIV primary cortical neurons from WT and *Cul3*^+/−^ mice, immunostained with MAP2 (green) and phalloidin-rhodamine (red); Scale bar is 25 μm; yellow triangles point to F-actin puncta on dendritic segments for better visualization. **d**, **e** Quantification of F-actin puncta and F-actin intensity on MAP2 positive dendrites, normalized by dendrite length. Symbols represent independent neurons and color represents littermates. Data are presented as mean ± SD (*n* = 4 per genotype, at least 7–10 neurons per mouse). Significance is calculated using two-tailed *t*-test; ***p < 0.001.

Neuron cytoskeleton is a complex and elaborate structure, with actin, microtubules and intermediate filaments playing dynamic and coordinated roles in determining neuron shape, movement, and connectivity during embryonic development. The cytoskeleton is critical for formation of specialized structures, such as growth cones, which are responsible for axon and dendrite elongation and guidance during development, synaptic boutons and dendritic spines, which form the structural basis for neural communication. Proteomic profiling of mouse brain pointed to neuronal cytoskeleton as one of the cellular structures impacted by *Cul3* haploinsufficiency. We found intermediate filament proteins to be upregulated across multiple DEP datasets in *Cul3*^+/−^ mouse (Fig. 5a), along with upregulated embryonic CX module *8pink* containing these proteins (Fig. 5c and Supplementary Table S11). At the same time, proteins involved in actin cytoskeleton-related processes (actin filament binding, actin cytoskeleton organization, regulation of actin cytoskeleton polymerization, regulation of actin filament length) were found to be downregulated in *Cul3*^+/−^ brain. Specifically, we identified significantly downregulated *2blue* module in the embryonic and *7black* in the adult CX modules with actin cytoskeleton-related GO functions (Fig. 7a, b and Supplementary Table S11).

To validate potential actin cytoskeleton defects in *Cul3*^+/−^ mouse, we co-stained 21DIV cultured mature primary cortical neurons with Phalloidin-rhodamine and mature neuron marker Map2 to quantify filamentous actin (F-actin) (Fig. 7c). We observed significant reduction of Phalloidin intensity in *Cul3*^+/−^ mutant neurons (*P* < 0.01, two-tailed *t*-test, Fig. 7d), as well as reduction of the number of F-actin puncta per dendrite length (*P* < 0.001, two-tailed *t*-test, Fig. 7e). Dendrites shortening and filamentous actin loss could have contributed to the defects in neural network activity observed in the mutant mice by MEA.

### RhoA inhibition rescues dendritic growth and network activity of cortical neurons

**Fig. 8:**
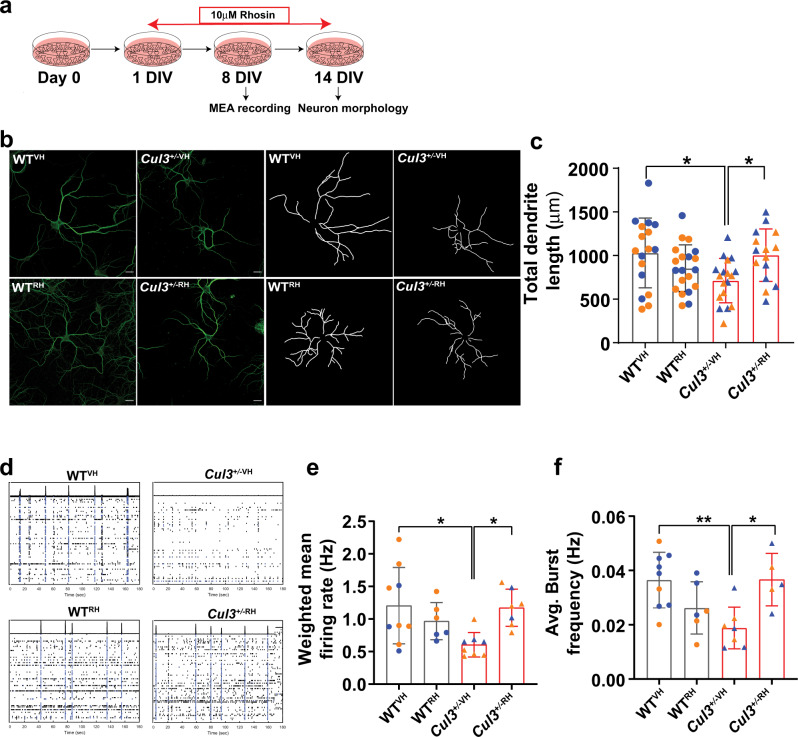
RhoA inhibition rescues dendritic growth and network deficits. **a** Flow diagram showing treatment timeline of primary cortical neurons with RhoA inhibitor Rhosin (RH). Half of the media containing Rhosin or vehicle was replaced every third day until the day of experiments. **b** Representative images of 14DIV primary cortical neurons (left panel) and tracings (right panel); Scale bar is 25 μm. The vehicle (Vh) and Rhosin (Rh) treated cells were immunostained with Map2 and dendrite tracing was performed. **c** Rhosin treatment rescues decreased dendrite length phenotype in *Cul3*^+/−^ neurons. Symbols represent independent neurons and color represents littermates. Data are presented as mean ± SD (*n* = 2 per genotype, at least 8–10 neurons per mouse). Significance is calculated using One-way ANOVA and Tukey test for multiple comparison; *p < 0.05. **d** Representative raster plots of spontaneous spike activity from 8DIV primary cortical neurons. **e** Spontaneous spike activity is significantly reduced in *Cul3*^+/−^ cortical neurons and rescued by treatment with 10 µM Rhosin; *p < 0.05, One way ANOVA and Tukey test for multiple comparison. **f** Average burst frequency is significantly reduced in *Cul3*^+/−^ neurons and rescued by treatment with 10 µM Rhosin; *n* = 6–9 mice per genotype, **p < 0.01; *p < 0.05, One-way ANOVA and Tukey test for multiple comparison was used.

Since RhoA regulates neuronal actin cytoskeleton remodeling and neurite outgrowth, and we found RhoA upregulated in *Cul3*^+/−^ mice, we investigated whether RhoA inhibition could rescue decreased dendritic length and neural activity phenotypes in primary cortical neurons. We took advantage of a previously described pharmacological inhibitor of RhoA activity, Rhosin. Rhosin was shown to specifically block RhoA activation and induce neurite outgrowth [25]. We repeated neuron morphometric analysis and used Rhosin (RH) or vehicle (VH) to treat primary cortical neuronal culture from 2 DIV until 14 DIV (Fig. 8a). We replicated decreased dendritic length phenotype in VH-treated *Cul3*^+/−^ neurons, and Rhosin treatment rescued dendritic length defects in *Cul3*^+/−^ to the level indistinguishable from the WT (*P* < 0.05, One-way ANOVA, Fig. 8b, c). Next, we measured network activity of primary cortical neurons at DIV8 with MEA in the presence and absence of Rhosin. We observed that Rhosin treatment was able to rescue the reduced weighted mean firing rate (*P* < 0.01, One-way ANOVA) and burst activity (*P* < 0.05, One-way ANOVA), returning these parameters to the levels comparable with vehicle-treated WT cortical neurons (Fig. 8d–f).

To understand the impact of prolonged Rhosin treatment, we conducted another MEA experiment, where we have plated cortical neurons at low density and recorded the activity for a longer period of time, up to day 19. At day 4, no network activity was observed (Supplementary Fig. S15). We began to observe network activity by day 8, and it continuously increased through day 19. Burst frequency followed a similar trend, except that it started to decrease after day 16. The reduction in weighted mean firing rate and in burst frequency between WT and *Cul3*^+/−^ were consistent throughout the recording. Thus, we recapitulated our earlier finding (Fig. 6f, g), and observed significant differences between WT^VH^ and Cul3^VH^ on days 8 and 12, with the trend in the correct direction on days 16 and 19. Interestingly, the treatment with Rhosin was able to rescue network activity on day 8, but not at the later time points. This points to a potentially narrow therapeutic window for Rhosin treatment. In summary, we demonstrated that Cul3 haploinsufficiency dysregulates neuron cytoskeleton and neurogenesis through increased active RhoA levels during cortical neuron development. The upregulation of active RhoA could be responsible for cellular, molecular and general neurodevelopmental defects observed in the *Cul3*^+/−^ mutant mice.

## Discussion

Developmental dysregulation of autism risk genes could affect a variety of neurobiological processes leading to anatomical and behavioral phenotypes in animal models and humans. However, the molecular mechanisms behind the majority of autism-associated mutations remain unknown. Here, we identify cellular and molecular processes dysregulated as a result of *Cul3* haploinsufficiency using a genome-engineered CRISPR/Cas9 mouse model. We provide evidence that defects in neuron cytoskeletal proteins and cortical neurogenesis along with RhoA signaling are potential mechanisms of *Cul3* haploinsufficiency. Our findings could have translational implications for the carriers of *Cul3* gene-disrupting variants.

The observed profound changes at the neuroanatomical level suggest that the impact of *Cul3* mutation on the brain begins early in development. Specifically, we found decreased cortical thickness and reduced density of TBR1-positive neurons at a very early developmental time point, at the embryonic day 14, implicating altered neurogenesis. The neurogenesis defects then persist into adulthood, as we also detect reduction of the NeuN- and BRN-positive layer II–IV neurons in the adult *Cul3*^+/−^ cortex. There are several mechanisms that could be implicated in the observed phenotypes, including altered proliferation, differentiation, migration, and/or increased apoptosis. We did not observe altered progenitors density or proliferation defects during mid-corticogenesis at E14, but we could not exclude the possibility that one or both of these processes could be disrupted at a later developmental stages. The reduction in TBR1-positive neurons points towards potentially defective neuronal differentiation and generation, and we also observed increased apoptosis of cortical neurons at later stages. Collectively, many different processes are likely contributing to the observed phenotypes.

Abnormalities in dendritic outgrowth and neuronal connectivity between the higher-order association areas have been considered as some of the major defects in ASD [23, 26]. Our proteomic analyses identified module of co-expressed proteins with neuron projection development functions. The observed dendritic growth deficits of *Cul3*^+/−^ neurons are likely influencing synaptic connectivity and network activity, as we observed reduced firing rate and bursting frequency of these neurons. RhoA is one of the proteins that controls neurite outgrowth, and RhoA upregulation was previously implicated in loss of spines and neurite retraction [10, 11, 14, 25, 27]. Previously developed genetic autism mouse models (SETD5, UBE3A, SHANK3, Timothy syndrome) also observed reduced neurite outgrowth, sometimes in concert with the reduced synaptic connectivity [27–30]. The iPSC-derived neurons from Timothy syndrome patients also exhibited dendrite retraction phenotype [27]. Thus, reduced neurite outgrowth in *Cul3*^+/−^ cortical neurons, possibly due to upregulated RhoA signaling, could be a shared phenotype among different autism models, including *Cul3*^+/−^.

Dysregulation of neuron cytoskeletal proteins, in particular, suggests a possible mechanism underlying *Cul3* haploinsufficiency. Neuronal cytoskeleton is crucial for a number of biological functions such as extracellular matrix organization, cell adhesion, microtubule formation, neuron migration, motility and locomotion, and cilium-related functions. We observed all of these processes to be enriched by either transcriptome or proteome profiling of *Cul3*^+/−^ brain. In addition, neuron cytoskeleton is essential for proper neurogenesis, branching, dendrite arborization and synapse formation [31]. Cul3 impacted a number of cytoskeletal proteins, including Ina, Vim, and Pls3. Intermediate filaments (i.e., Ina) play important role in promoting microtubule stabilization, and their accumulation results in hyperstabilization of microtubules, leading to disruption of synaptic vesicle transport [32]. Vimentin (Vim) is known to be involved in early neuron outgrowth and cell polarity, and acts as a template for microtubule stabilization during cell migration [33]. The increased neuronal apoptosis and defective neurite outgrowth may be the consequence of accumulated intermediate filaments that affect the transient actin and microtubule cytoskeleton [32]. We experimentally confirmed loss of filamentous actin puncta in *Cul3*^+/−^ neurons, providing further evidence for cytoskeletal defects.

The Rho GTPases play essential roles in regulating neuronal morphology and function. At least 20 high-risk autism genes encode Rho GTPase regulators or effectors [34]. RhoA also plays an important role in regulating actin remodeling during early brain development [35]. We attribute observed cytoskeletal and neuronal defects in *Cul3*^+/−^ mice to the upregulation of RhoA signaling, since we observed upregulated RhoA in embryonic and adult cortex of *Cul3* mutant mice. We also were able to rescue dendrite length and network connectivity phenotypes by pharmacological inhibition of RhoA activity with Rhosin. Interestingly, Rhosin treatment was able to rescue network activity on day 8, but not at the later time points. One possible explanation could be that RhoA plays a crucial role in early neuron growth and migration [36] and thus, RhoA inhibition was able to improve early network activity. After day 8, other cellular processes such as spine maturation and establishment of the synaptic connectivity begin, which may have interfered with the rescue. Our results point to potentially a narrow therapeutic window for Rhosin, at least with regards to rescue of these specific phenotypes. It would be interesting to investigate whether Rhosin can rescue the behavior or brain anatomical defects. In addition, Cul3 could also dysregulate other pathways upstream or downstream of RhoA, which may impact other phenotypes.

*Cul3*^+/−^ mice share similarities with other autism mouse models with regards to brain volumetric changes [37]. Interestingly, the Cul3 model is most similar to the group comprised of 16p11.2, Mecp2, and CNTNAP2 models. Comparable brain structural changes were also reported in a different 16p11.2 deletion mouse model [38], as well as in the TAOK2 mouse model [39]. The similarities between 16p11.2 and Cul3 models is not surprising, because one of the genes from 16p11.2 CNV region, *KCTD13*, is an adapter for Cul3 ubiquitin ligase. Thus, the same pathways may be dysregulated by the 16p11.2 CNV and Cul3 mutation. In addition, we compared our *Cul3*^+/−^ model with two recently published Cul3 conditional mouse models [40, 41]. These models were generated by either knocking down or knocking out Cul3 in excitatory neurons of prefrontal cortex [41], or by activating mutation at progenitor’s level, leading to complete elimination or haploinsufficiency in neurons and glia [40]. The homozygous germline knockout Cul3 mice were embryonically lethal ([8] and this study), therefore, we resorted our comparison to heterozygous mice from these studies with our heterozygous *Cul3*^+/−^ model.

Our *Cul3*^+/−^ mouse model differs from two other models in at least two ways: (1) CRISPR technology allowed us to generate germline mutation that is present in all cell types and in all brain regions and other tissues, which is more comparable to the human carriers; (2) Cul3 mutation is present starting from conception and from the very early developmental time points. Finally, none of two former models investigated the impact of Cul3 mutations in embryonic or early developmental periods, which is crucial due to the highest expression of Cul3 gene during early prenatal development. The changes observed at later developmental time points in the published studies may be just the consequences of early embryonic deficits. Interestingly, both studies reported reduced body weight only in Cul3 homozygous knockout mice, but not in heterozygous mutants, suggesting a milder phenotypes observed in the previous models compared to our CRISPR *Cul3*^+/−^ mouse. Dong et al. model also observed hyperactivity, in agreement with our model. Previous models did not perform transcriptomic analyses, therefore, we were able to only compare proteomics results from these models with our findings (Supplementary Fig. S16). Importantly, neither Dong nor Rapanelli studies detected differentially expressed proteins with FDR-corrected *p*-values in their proteomics data, because none of their proteins surpassed FDR correction. When genome- and proteome-wide studies are carried out, only genes/proteins with FDR-corrected *p*-values are normally reported to avoid spurious findings. We, on the other hand, have detected 2329 differentially expressed proteins in embryonic, 986 in early postnatal and 402 in adult cortex; and 1016 in embryonic, 252 in early postnatal and 5 in adult cerebellum with FDR < 15%. This suggests that our study had greater power to detect differentially expressed proteins and to implicate molecular pathways, potentially due to stronger effect of Cul3 mutation due to its germline transmission. Clearly, the embryonic period stands out with much greater number of detected DEPs. Most importantly, Dong and Rapanelli studies did not converge on a common molecular mechanism underlying the impact of Cul3 mutation. Dong et al. observed translational dysregulation in their Cul3 model, with the main contributing player translation initiation factor EIF4G1. In our study, we did not observe EIF4G1 to be differentially expressed at any period or in any brain region, although it has been detected by proteomics. At the same time, our upregulated DEPs Pls3 and Ina were also upregulated in Dong model with nominal uncorrected *p*-values. Rapanelli et al. study narrowed down to histone methyltransferase Smyd3. We have not detected Smyd3 in our proteomics experiments, therefore, we cannot validate this specific finding. However, Rapanelli also observed RhoA upregulation and reduced spine density, in agreement with our results. Interestingly, Pls3 was the only protein that was found to be upregulated in Dong, Rapanelli and in our study. We conclude that our study captures early molecular defects in cortical neuron cytoskeleton and neurogenesis that likely lead to molecular and physiological changes at later developmental time points observed by other models.

In conclusion, our study demonstrates that *Cul3* germline haploinsufficiency impairs early brain growth and development by dysregulating neuronal cytoskeleton and neural connectivity through a molecular mechanism related to RhoA signaling. Other pathways, detected later in development and in adulthood, are likely the result of these initial defects that begin during early embryogenesis. Going forward, it would be interesting to develop Cul3 patient-derived and isogenic CRISPR models, such as cortical organoids, to further investigate whether similar defects are observed in human Cul3 mutation carriers. As we have previously demonstrated [42, 43], transcriptional and electrophysiological phenotypes of cortical organoids resemble those of human fetal brains. Given early developmental defects evoked by the Cul3 haploinsufficiency, cortical organoids would serve as an attractive model for translating animal findings into human disease mechanisms.

## Materials and methods

### Generation of *Cul3*^+/−^ mice using CRISPR–Cas9 genome editing and maintenance

For generating *Cul3* mouse model with the patient-specific p.Glu246Stop (E246X) mutation [15], single-guide RNAs (sgRNA) and single-stranded DNA oligonucleotides (ssODN) were designed to create the E246X frameshift mutation. Two single guide (Sg) RNA, Sg1-GGATTAATGAGGAAATAGAG and Sg2- AGTAGAAGCTAGGATTAATG, with little off-target specificity for *Cul3* (NM 016716.5) were designed using http://crispr.mit.edu/ platform and CHOP-CHOP (https://chopchop.cbu.uib.no/). The specificity and efficiency of each sgRNA was validated using in vitro Cas9 cleavage assay. Generation of transgenic lines was performed by the UCSD Moores Cancer Center Transgenic Mouse Core Facility. Briefly, microinjections consisting of sgRNA1 (0.6 μM of crRNA, 0.6 μM tracrRNA), 0.5 μM of ssODN and 30 ng/μl Cas9 protein (PNA-Bio, USA) was injected in pronuclear stage embryos in the C57BL/6N genetic background. The exon-6 specific genotyping primers, *Cul3*-ex6-Fwd: CTGCATGAAATGTGTCTTTAACTGT and *Cul3*-ex6-Rev: CCACCCCACTGCTAACTAGG, were used for PCR genotyping followed by Sanger sequencing. The Heterozygous *Cul3* founder mouse harboring 1bp-insertion in exon6 was expanded via breeding with wild-type (WT) C57BL/6N mice. The carrier male *Cul3*^+/−^ mice were bred for at least four generations before starting any experiments to eliminate off-target effect of CRISPR. *Cul3* heterozygous (*Cul3*^+/−^) mice were viable, reached normal lifespan, and were fertile irrespective of sex. Animals were housed in groups of four animals per cage and kept on a 12 h light/dark cycle (lights on at 7:00 am), with food and water available *ad libitum*. Embryonic time points were determined by plug checks, defining embryonic day (E) 0.5 as the morning after copulation. No randomization was used, and blinding was used during animal behavioral analyses and the image analyses. All experiments were approved by the Institutional Animal Care and Use Committees (IACUC) of the University of California San Diego (protocol number-S17029).

### Analysis of CRISPR off-target genes

The sgRNA was designed to target a patient-specific mutation in *Cul3*. In order to reduce off-target effects, we used Cas9 protein, which have much shorter half-life (about 24 h in the cells) than Cas9-plasmid or Cas9-mRNA. In addition, we back-crossed mutation carrier animals for four generations with C57BL/6N animals to eliminate the remaining off targets. Two different software were used to predict off-target effects of sgRNA were used (https://chopchop.cbu.uib and https://crispr.cos.uni-heidelberg.de/). Top 10 off-target regions have mismatch of 3 base pairs or more, and they are present in exonic or intronic region of the genes, or and spliced ESTs sites (Supplementary Table S14). We also investigated the mRNA expression level of off-target genes by analyzing TPM values for the off-target genes in our transcriptomic data to make sure that the gene expression of the off-targets was not altered. We observed that only Cul3 was significantly downregulated in *Cul3*^+/−^ mice, and all other genes had similar expression levels in both WT and *Cul3*^+/−^ mice (Supplementary Fig. S17).

### Mouse behavioral testing

*Cul3*^+/−^ and WT littermates were used for behavioral experiments. Male and female mice were group housed post-weaning, segregated by sex, with unrestricted access to food and water, and were kept on a 12 h light/12 h dark cycle. The number of animals used in each behavior testing is mentioned in the figure legends. For all tests, 1 h of habituation to the room before each test was performed. *Cul3*^+/−^ and their WT littermates were evaluated on the same day and by the same equipment, equipment was cleaned with 70% ethanol after each animal. Testing for open field, social preference, object recognition, repetitive behavior and social behavior were conducted during the light phase (10 am–5 pm) on different days. All testing equipment was positioned inside a photo light box that have dim overhead fluorescent lighting (14 lux). Tests were performed starting with the least aversive to most aversive task, and either scored automatically or by an experimenter blind to the genotype.

#### Developmental milestones

Development of reflexes and growth was evaluated as previously described [44]. Reflexes were considered to be acquired only after they had been observed for 2 consecutive days.

#### Open field

The apparatus measured ~43 × 43 × 33 cm (width x depth x height), the center was defined as the middle 26 × 26 cm. Mice were tested in a white open field for a total of 30 min per animal. The behavior and time spent in the center vs boundaries was recorded for 30 min by video camera. The videos were analyzed by MATLAB software and scored for 10, 20, and 30 min.

#### Novel object recognition

On day 1 of the experiment, mice were familiarized with the training room (1 h) in their home cages. During the first day, the mice explored an empty chamber for 5 min (plastic chamber, 61 × 42 × 22 cm). On testing day, the animals were placed in the testing chamber for 1 min to re-habituate. Then two identical objects (plastic figures) were placed into the testing chamber. The mouse was placed in the chamber for 15 min to explore the objects. After a 3 h delay, the mouse was placed in the testing chamber with one object identical to the previous object, and a new object with different shape, texture, and color. Over a 15-min time period, the relative amount of time the animal spends exploring the new object compared to the familiar one was quantified by MATLAB software.

#### Repetitive behavior

Each mouse was placed in a novel test cage with no bedding or food. The animal spent a total of 20 min in novel test cage (10 min for habituation, 10 min for observation). The last 10 min of repetitive behavior was scored for the following categories: self-grooming, circling, isolation, or another unusual behavior. The scoring was done manually by an experimenter blind to the genotype.

#### Social preference test

The three-chamber test was done in a social box and grids were purchased from Harvard Apparatus (Catalog #76-0673, #76-0674). The test was performed as previously described [45]. One day before testing mice were habituated to the three-chamber apparatus, and were allowed to explore the apparatus for 10 min, with empty grid at each side of the chamber. A plastic dish was placed on top of each grid to prevent climbing to the top. On test day, the test mouse was then placed in the center chamber and was free to explore the chambers for 10 min in each of the following phases: the first phase two identical non-social stimuli (Red lego blocks); the second phase non-social stimulus (yellow, gray and green Lego Blocks), and a social stimulus (age and sex matched mouse); the third phase familiar mouse and a novel mouse. The familiar mouse is the same mouse used in the second phase. The chamber was cleaned with 70% ethanol between phases. The chamber time and sniffing time was scored for the intervals of 10 min. Manual (blind analysis) and automated (MATLAB Software) methods of scoring chamber time and cylinder sniffing were used.

### Magnetic resonance imaging (MRI) of adult and early postnatal brains

The cohort used for behavioral testing was subsequently used for adult brain MRI study.

#### Perfusion

Mice undergone behavioral testing (*Cul3*^+/−^: 10 male, 10 female; WT: 8 male, 10 female) were anesthetized with a ketamine/xylazine mix (10 mg/kg) and intracardially perfused with 30 mL of 0.1 M PBS containing 10 U/mL heparin (Sigma) and 2 mM ProHance (a Gadolinium contrast agent), followed by 30 mL of 4% paraformaldehyde (PFA) containing 2 mM ProHance. After perfusion, mice were decapitated and skin, lower jaw, ears and cartilaginous nose tip were removed as previously described described [46, 47]. The brain within the skull was incubated in 4% PFA containing 2 mM ProHance overnight at 4 degrees Celsius then transferred to 0.1 M PBS containing 2 mM ProHance and .02% sodium azide for at least 7 days prior to MRI scanning.

#### Imaging

After perfusion, a multichannel 7.0 Tesla MRI scanner (Agilent Inc., Palo Alto, CA) was used to image the brains within their skulls. Sixteen custom-built solenoid coils were used to image the brains in parallel. In order to detect volumetric changes via anatomical imaging, the following parameters were used: T2-weighted, 3-D fast spin-echo sequence, with a cylindrical acquisition of k-space, a TR of 350 ms and TEs of 12 ms per echo for 6 echoes, field-of-view equal to 20 × 20 × 25 mm^3^ and matrix size equal to 504 × 504 × 630 mm^3^. These parameters output an image with 0.040-mm isotropic voxels. The total imaging time was 14 h.

#### Analysis

All images were registered through iterative linear and nonlinear registrations to create a consensus average brain image. Deformation fields were computed, which describe the differences between each individual and the average. The Jacobian determinants of the deformation fields were then calculated as measures of volume at each voxel [48]. Structure volume was also calculated by warping a pre-existing classified MRI atlas onto the population atlas, which allowed for the calculation of 182 structures [49–51]. Multiple comparisons were controlled for using the false discovery rate (FDR) [52].

### Young mice (P7)—56 μm DTI

#### Perfusion

For younger mice, a similar perfusion protocol was followed with minor modifications. 15 ml instead of 30 ml of perfusion solution was used at both stages of the intracardial perfusion. Additionally, the skin and other skull structures were not removed following decapitation to prevent possible damage to the skull and brain of the neonatal mouse.

#### Imaging

For younger brains, diffusion tensor imaging was used to optimize gray/white matter contrast, as brains have yet to undergo large scale myelination in development. The diffusion sequence is a 3D diffusion-weighted FSE, with TR = 270 ms, echo train length = 6, first TE = 30 ms, TE = 10 ms for the remaining 5 echoes, one average, FOV = 25 mm × 14 mm × 14 mm, and a matrix size of 450 × 250 × 250, which yielded an image with 56 μm isotropic voxels. One b = 0 s/mm 2 image is acquired and 6 high b-value (b = 2147 s/mm 2) images were acquired at the following directions (1,1,0), (1,0,1), (0,1,1), (−1,1,0), (−1,0,1) and (0,1,−1) corresponding to (G x,G y,G z). Total imaging time was ~14 h.

#### Analysis

First, the six high b-value images were averaged together to make a high contrast image necessary for accurate image registration. Then, all average high b-value images were linearly and nonlinearly registered together to create a consensus average brain. Deformation fields, which describe the deformations needed to take each individual mouse anatomy into the consensus average space, were calculated, and the Jacobian determinant of those deformation fields were then calculated as measures of volume at each voxel. Structure volume was also calculated by warping a pre-existing classified MRI atlas onto the population atlas, which allowed for the calculation of 56 structures. Multiple comparisons were controlled for using the false discovery rate (FDR) [52].

To calculate the change in region-specific volumes for a larger regions such as somatosensory cortex, cerebellum, and dentate gyrus (Fig. 2e), the sum of the volumes of all sub-regions comprising each of these larger regions was calculated for each animal. Then, the mean, standard deviation, effect size and % difference were calculated for WT and *Cul3*^+/−^ groups. The two-tailed *t*-test was used for comparing the statistical differences.

### Immunohistochemistry

Mice were deeply anesthetized by xylazine and ketamine and were perfused with 4% paraformaldehyde (PFA). Whole-brain tissues were extracted and dehydrated in a 30% sucrose solution at 4 °C overnight. The 30 μm thick brain slices were prepared using a cryostat (Leica, Germany). Slices were rinsed in 0.01 M PBS, permeabilized with 0.1%TritonX-100 in PBS and blocked in 3% FBS (with 0.1% Triton X-100) for 1 h. Primary antibody against NeuN (mouse 1:1000, Millipore, USA), BRN2 (rabbit 1:1000, Cell Signaling Technology, USA) was added for overnight incubation at 4 °C. Donkey anti-rabbit Alexa 555 and anti-mouse Alexa 488 secondary antibody (Life Technology, Camarillo, CA, USA) was added at room temperature for 2 h incubation. After PBS rinsing, DAPI was added for nuclear staining. Slices were mounted using ProLong Gold antifade mountant (Invitrogen). Fluorescent images were taken on Leica SP8 confocal microscope using ×10 inverted objective and quantification was done using ImageJ (NIH). To examine cortical layering, the length of the layer containing NeuN-positive cells within the somatosensory cortex was measured (*N* = 6 littermate animals per genotype). For the quantification of mature neurons in layer II–IV, the number of total NeuN-positive cells and BRN2-positive cells in II–IV cell layers was performed within the somatosensory cortex and normalized to the area used (*N* = 6 littermate animals per genotype).

### Immunofluorescent staining of E14 embryonic cortical slices

E14 mice heads were decapitated and drop fixed in 4% PFA overnight. After overnight fixation, heads were washed with PBS to remove PFA and dehydrated in 30% sucrose for 24–48 h. The heads were embedded in O.C.T. (Tissue Tek) and 18 μm sections were prepared on a cryostat (Leica, Germany). For assessment of the cellular composition of the embryonic cortex, immunofluorescent staining was performed as described above. The following primary antibodies were used: anti-SOX2 (rabbit, 1:200, Cell Signaling), anti-Ki67 (rabbit, 1:200 Abcam), and anti-TBR1 (rabbit, 1:200, Millipore). For the quantification, the number of SOX2, Ki67, and TBR1-positive cells were counted in individual cortex sections and normalized to the area used (*N* = 4 littermate animals per genotype).

### Primary cortical neuron culture

Cortices were dissected from WT and *Cul3*^+/−^ mouse embryonic brains at E17.5. Cortex from each brain was cultured individually. Dissociation was initiated by incubating the dissected cortices in 1 ml of Accumax (Innovative Cell Technologies Inc) for 30 min at 37 °C followed by 5 min incubation in 10 mg/ml DNaseI (Sigma Aldrich), the dissociated cells were gently triturated with fire-polished glass Pasteur pipette to make single cell suspension, and then passed through 40-micron nylon filter to remove any non-dissociated tissue. Cells were counted with cell counter (BioRad) and 1 × 10^5^ cells were seeded onto glass coverslip coated with 0.01% P-L-ornithine and 5 μg/mL mouse natural laminin in 24-well plates. The plating media contained Neurobasal medium, 2% B27 supplement, 10% horse serum, 1% penicillin/streptomycin, and 2 mM L-glutamine (Invitrogen). After 12 h, media was changed to serum-free feeding media containing Neurobasal medium, 2% B27 supplement, 1% penicillin/streptomycin, and 2 mM L-glutamine. At DIV2-4, cultures were treated with 1 mM Cytosine b-D-arabinofuranoside hydrochloride (Ara-C) (Sigma Aldrich) to inhibit glial cell proliferation. The cells media were half replaced with new media containing AraC every 4 days. Cultures were maintained at 37 °C and 5% CO_2_. All media components were from GIBCO unless otherwise specified.

### Immunocytochemistry

Coverslips containing primary cortical neurons were fixed in 4% paraformaldehyde (PFA) for 15 min and washed three times with PBS. Permeabilization and blocking was performed with 3% bovine serum albumin (BSA, Sigma-Aldrich), 0.1% Triton X-100 (Sigma Aldrich) in PBS for one hour at room temperature. The coverslips were then incubated overnight at 4 °C with primary antibodies diluted in solution containing 3% BSA. PBS was used to wash the primary antibodies and the coverslips were incubated with secondary antibodies in solution containing 3% BSA for 1.5 h at room temperature. The following primary antibodies were used for immunostaining: MAP2 (rabbit 1:1000, Cell Signaling Technology); Rhodamine Phalloidin (Cytoskeleton). Alexa Fluor Dyes (Abcam) were used at 1:1000 dilution as secondary antibodies. Nuclei were visualized with DAPI (1:25,000, Life Technologies). Slides were mounted using ProLong Gold antifade reagent (Invitrogen) and acquired using Leica SP8 confocal microscope with oil-inverted 40x objective. Image analysis was performed with ImageJ software

### Morphological analysis

Images were processed and analyzed with ImageJ 1.5 software. For soma area calculation, the perimeter of the Map2-positive cell bodies were manually outlined and measured. For total dendrite length analysis, the simple neurite tracer plugin for ImageJ 1.5 was used. Map2-positive neurites from each neuron were traced and the dendrite length was calculated by adding individual lengths for every neuron. F-actin positive puncta on dendrites was calculated by visually counting all protrusions from a dendrite within a 15–25 mm distance starting at a secondary branch point. One to three dendritic segments were analyzed per neuron. The number of F-actin puncta was normalized by dendritic length, similar region were also used for quantification of F-actin intensity using Image J 1.5. Image analysis and quantification was performed by the trained experimenter blind to the genotype.

### Multi-electrode array (MEA) recordings

Primary cortical neurons from WT and *Cul3*^+/−^ E17.5 embryos were cultured on 12-well Axion Maestro multielectrode array (MEA) plates (Axion Biosystems, Atlanta, GA, USA). Each 12-well MEA plate from Axion Biosystems was coated with 100 μg/mL poly-L-ornithine and 10 μg/mL laminin for 24 h before seeding. 1.5 × 10^6^ neurons were plated on the coated MEA plates and a half-medium change was performed every other day using neurobasal+B27 feeding medium. Spontaneous spike activity was recorded on 8 DIV. Recordings were performed at 37 °C using a Maestro MEA system and AxIS Software Spontaneous Neural Configuration (Axion Biosystems). Briefly, Spikes were detected with AxIS software using an adaptive threshold and then analyzed using Axion Biosystems’ Neural Metrics Tool. Spike time stamps were exported to Neuroexplorer (Axion Biosystems) for the creation of raster plots. Bright-field images were captured to assess cell density and electrode coverage. Quality control included: (1) equal plating of cortical neurons from both genotypes; (2) the threshold for MEA activity was set to 5 spikes/min during data analysis, meaning that the electrodes that do not meet this minimum threshold criteria were eliminated from the analysis. This ensures that only electrodes covered by the live, actively firing neurons are considered in the analyses; (3) cell imaging to ensure comparable coverage of MEA plate; (4) protein quantification after completion of recordings to ensure equal well coverage between genotypes (Supplementary Fig. S15, Supplementary Table S15).

### Pharmacological treatment of cortical neurons with Rhosin

For morphological phenotype rescue experiments, cultured cortical neurons were grown in Rhosin-treated neurobasal media. Rhosin (Tocris) was added to the final concentration of 10 µm to the media at 2 DIV stage. The same amount of Rhosin was added every 4 days during all subsequent media changes. The cells were grown for 14 days, at which dendrite staining was carried out with Map2 antibody as mentioned in the immunocytochemistry section. An equivalent amount of vehicle (dimethylsulfoxide, DMSO) was added to a final concentration of 0.001% to growth media to obtain vehicle-treated WT and Cul3^+/−^ cortical neurons.

For phenotype rescue experiments at day 8, the cells were plated on MEA plates as described above and media changes were performed every 3rd day. The same amount of Rhosin was added at 1 DIV stage and at all subsequent media changes. The cells were grown for 8 days, at which MEA recording and image acquisition was carried out. After completion of the experiment the cells were extracted and lysed to quantify total protein in each well.

For prolonged MEA cortical neuron recordings and phenotype rescue, 2 × 10^5^ neurons were plated on the coated 12 well MEA plates, and a half-volume feeding medium (neurobasal+B27) change was performed every 3rd day. The 10 µM Rhosin was added on 1 DIV stage and at all subsequent media changes. Spontaneous spike activity was recorded on 4, 8, 12, 16, and 19 DIV. Recordings and analysis were performed as described in the above MEA section.

### TUNEL assay

We used a terminal deoxynucleotidyl transferase (TdT) dUTP nick-end labeling (TUNEL) assay to measure the apoptosis of the primary cortical neurons. The assay was performed using APO-BrdU TUNEL assay kit (Invitorgen) as described by the manufacturer. In brief, primary cortical neurons were fixed with 4%PFA followed by O/N incubation in 70% ethanol at −20 °C followed by PBS washing. A freshly prepared TUNEL reaction buffer (50 μL per sample) was added at 37°C incubation for 1 h. After rinsing with the rinse buffer, the Propidium Iodide (PI) was used for nuclei staining. The cells were acquired in BD accuri C6 (BD, Franklin Lakes, NJ) flow cytometer. All the acquired cells were considered as population of interests for further analysis. Respective single color-stained samples were used to correct color compensation overlapping between two channels. The percentage of TUNEL-positive and PI-positive cells were analyzed with FlowJo software (FlowJO LLC, Ashland, OR) with respect to the corresponding unstained samples. The percentage change in dual positive cells, i.e., PI- and BrdU-488 positive cells was calculated.

### RNA isolation for bulk RNA sequencing and qPCR

Total RNA was extracted at three developmental time periods (embryonic E17.5, early postnatal (day 7) and adult (4–6 weeks)) from three brain regions (cerebral cortex, hippocampus, and cerebellum) of WT and *Cul3*^+/−^ mice using the QIAGEN RNAeasy isolation kit (QIAGEN) following manufacturer’s instructions. RNA sequencing was performed using equal input amount of total RNA for each sample. RNA samples were ribodepleted using Ribo-Zero rRNA Removal Kit (Illumina) and library preparation was performed using the True-Seq Stranded Total RNA kit for Illumina Sequencing according to the manufacturer’s instructions. Paired-end RNA sequencing (2 × 150 bp) was performed on an Illumina HiSeq4000 to an average depth of 40 M reads per sample.

For Quantitative RT-PCR (qPCR) experiments (Fig. 1e), cDNA was synthesized starting from 100 ng of total RNA with the SuperScript III First-Strand Synthesis kit and oligo dT (Invitrogen). The qPCR was performed using the CFX96 Touch™ Real-Time PCR Detection System (Bio Rad) using Power SYBR Green PCR Master Mix (Applied Biosystems). The following primers were used for Cul3—Cul3_Fwd: TCAAACAGTTGCAGCCAAAC and Cul3_Rev: GAATCGAGCCTTCAGTTGCT. GAPDH was used as housekeeping gene for the normalization, the following primers were used for GAPDH—GAPDH_Fwd: AGGTCGGTGTGAACGGATTTG and GAPDH_Rev: TGTAGACCATGTAGTTGAGGTCA. Fold change in expression was calculated using the ΔΔ^Ct^ method. Data are presented as levels of Cul3 normalized to GAPDH levels.

### RNA-seq data processing pipeline

All 108 FASTQ files (36 embryonic, 36 early postnatal and 36 adult) (Supplementary Fig. S7) were run through a unified paired end RNA-Seq processing pipeline. Pipeline source code can be found on https://github.com/IakouchevaLab/CUL3. All fastqs were trimmed for adapter sequence and low base call quality (Phred score <30 at ends) using Cutadapt (v1.14). Trimmed reads were then aligned to the GRCm38.p5 (mm10**)** reference genome via STAR (2.5.3a) using comprehensive gene annotations from mouse Gencode (v16). Gene-level quantifications were calculated using RSEM (v1.3). Quality control metrics were calculated using RNA-SeQC (v1.1.8), featureCounts (v1.6.), PicardTools (v2.12), and Samtools (v1.3) (Supplementary Table S16, Supplementary Fig. S8).

### RNA-seq quality control, normalization, and differential gene expression analysis

RNA-Seq quality control and normalization expected counts were compiled from gene-level RSEM quantifications and imported into R for downstream analyses. Expressed genes were defined as genes with TPM > 0.1 in at least 80% of samples from each genotype (WT and *Cul3*^+/−^). A total of 17,363; 18,656; and 18,341 expressed genes from embryonic cortex, cerebellum, and hippocampus, respectively; 18,921; 18,276; and 18,396 expressed genes from early postnatal cortex, cerebellum, and hippocampus, respectively; and 18,061; 17,835; and 17,654 expressed genes from adult cortex, cerebellum, and hippocampus, respectively, were used in the downstream analysis using the mouse Gencode (v16) annotation gtf file. Only expressed genes were included into the analysis.

To account for possible batch effects, in the sex-blind differential gene expression analysis, batch effect removal was performed using EDASeq R package [53] and “remove unwanted variation” (RUVs) package in RUVseq [54]. Initial normalization of count data were done using upper quartile normalization [53], followed by RUVseq. The parameter k in RUVseq was set to the minimum value that separated the samples based on genotypes on the PCA plot (maximum k = 10). RUVs with different k values ranging from 3 to 8 were used after defining groups based on genotype (i.e., WT vs *Cul3*^+/−^). Differential gene expression analysis was performed with edgeR [55] using the negative binomial GLM approach and by considering design matrix that includes both covariates of interest (Genotype and Gender) and the factors of unwanted variation. Genes with a false discovery rate (FDR ≤ 0.1, Benjamini–Hochberg multiple testing correction) were considered significant and used for further downstream processing and analysis. To determine the sex effect, the differential expression analysis was performed for males and females separately using the same approaches.

Differentially expressed gene datasets for all regions (CX, CB, and HP) across individual developmental periods, and all periods (E17.5, P7, and P35) across individual brain regions, were considered for period- and region-wise *P*-value combination analysis. We performed a total six *P*-value combination analysis that included both, period- and region-wise comparisons. Preprocessing of the DEG datasets was done by selecting differentially expressed genes with similar trend (i.e., either up- or downregulated in mutant vs WT) based on log fold change expression, for respective regions-wise or period-wise comparisons. The Fisher *P*-value combination method with metaRNASeq R package [56] was used to combine the *P*-values for these sets of genes, following similar trend in the log fold change expression for each comparison. The combined FDR-corrected *P*-value for each set of genes was obtained using Benjamini–Hochberg method. Genes with combined FDR-corrected *P*-value of ≤0.1 in each region- and period-wise comparison were hierarchically clustered. We obtained a total 21 clusters from period- and region-wise analyses combined. GO terms enrichment was performed using gProfiler [57] for all gene clusters, resulting in around 1700 GO terms. Log fold GO enrichment was performed for all GO terms, as well as for shared by two or more clusters GO terms. These shared GO terms were subsequently hierarchically clustered to produce log fold GO term enrichment Figure (Fig. 4d).

### Gene ontology enrichment analysis

Enrichment of gene ontology terms (GO) biological process (BP) and molecular function (MF) was performed using gProfiler R package [57]. Background was restricted to the expressed set of genes by period (embryonic, early postnatal, adult) or region (cortex, cerebellum, and hippocampus) whenever appropriate. Ordered query was used, ranking genes by FDR-corrected *P*-value.

### Enrichment analysis of ASD-relevant gene sets

Enrichment analyses were performed using several established, hypothesis-driven gene sets including syndromic and highly ranked (1 and 2) genes from SFARI Gene database (https://gene.sfari.org/database/gene-scoring/); pre- and post-synaptic genes from SynaptomeDB [58]; genes with loss-of-function intolerance (pLI) > 0.99 as reported by the Exome Aggregation Consortium [59]; highly constrained genes [60]; and FMRP targets [61]. Fisher exact test was used to calculate the enrichment of significantly differentially expressed genes for each curated gene set. The background lists were the union of all analyzed genes. Significance values of the results were corrected for multiple hypothesis testing using Benjamini–Hochberg method.

### Quantitative TMT-mass spectrometry

#### Sample preparation

TMT mass-spectrometry experiments were performed on the WT and *Cul3*^+/−^ cerebral cortex and cerebellum at three developmental periods (embryonic, early postnatal, and adult). Tissues were collected from the remaining half of hemisphere of the brains used for RNA-seq experiments (except for embryonic and adult cerebellum), and snap-frozen in liquid nitrogen. After collection of the entire set, the tissues were lysed in RIPA buffer (20 mM Tris, pH 7.4, 150 mM NaCl, 1 mM EDTA, 1% sodium deoxycholate and 1% Triton X-100) supplemented with 1×EDTA-free complete protease inhibitor mixture (Roche) and phosphatase inhibitor cocktails-I, II (Sigma Aldrich). The lysates were centrifuged at 16,000 × *g* at 4 °C for 30 min, and the supernatants were collected. Protein concentration was quantified by modified Lowry assay (DC protein assay; Bio-Rad). Lysates were subjected to methanol-chloroform precipitation, resuspended in 8 M urea in 50 mM TEAB, reduced (10 mM TCEP at room temperature for 25 min) and alkylated (50 mM chloroacetamide at room temperature in the dark for 20 min). After another round of methanol-chloroform precipitation, pellets were dissolved by adding 6 M urea in 50 mM TEAB, and the protein concentration was estimated by BCA assay (Thermo, 23225). LysC/Tryp (Promega, V5073) was added by 1:25 (w/w) ratio to the proteins. After 3–4 h of incubation with 850 rpm shaking at 37 °C, reaction mixture was diluted with 50 mM TEAB for urea to be less than 1 M. After the o/n digestion, peptide concentration was estimated by colorimetric peptide BCA assay (Thermo, 23275), and the peptides were labeled with TMT 10-plex reagents (Thermo, 90110) for one hour, followed by 15 min quenching with hydroxylamine according to the manufacturer’s protocol. Equal amounts of reaction mixtures for each channel were pooled together and dried using SpeedVac. Dried peptides were resuspended in 0.1% TFA and fractionated using Pierce™ High pH reversed-phase peptide fractionation kit (Thermo, 84868) and then dried in SpeedVac.

#### Mass spectrometry

Each fraction was dissolved in buffer A (5% acetonitrile, 0.1% formic acid) and injected directly onto a 25 cm, 100 μm-ID column packed with BEH 1.7 μm C18 resin (Waters). Samples were separated at a flow rate of 300 nl/min on nLC1000 (Thermo). A gradient of 1–30% B (80% acetonitrile, 0.1% formic acid) over 160 min, an increase to 90% B over another 60 min and held at 90% B for a final 20 min of washing was used for 240 min total run time. Column was re-equilibrated with 10 μL of buffer A prior to the injection of sample. Peptides were eluted directly from the tip of the column and nanosprayed directly into the mass spectrometer Orbitrap Fusion by application of 2.8 kV voltage at the back of the column. Fusion was operated in a data dependent mode. Full MS1 scans were collected in the Orbitrap at 120 K resolution. The cycle time was set to 3 s, and within this 3 s the most abundant ions per scan were selected for CID MS/MS in the ion trap. MS3 analysis with multi-notch isolation (SPS3) [62] was utilized for detection of TMT reporter ions at 60 K resolution. Monoisotopic precursor selection was enabled, and dynamic exclusion was used with exclusion duration of 10 s.

#### Protein identification and quantification

Tandem mass spectra were extracted from the.raw files using Raw Converter [63] with monoisotopic peak selection. The spectral files from all fractions were uploaded into one experiment on Integrated Proteomics Applications (IP2, Ver.6.0.5) pipeline. Proteins and peptides were searched using ProLuCID [64] and DTASelect 2.0 [65] on IP2 against the UniProt reviewed Mus musculus protein database with reversed decoy sequences (UniProt_mouse_reviewed_contaminant_05-25-2018_reversed.fasta from IP2 standard database). Precursor mass tolerance was set to 50.0 ppm, and the search space allowed both full- and half-tryptic peptide candidates without limit to internal missed cleavage and with a fixed modification of 57.02146 on cysteine and 229.1629 on N-terminus and lysine. Peptide candidates were filtered using DTASelect parameters of -p 2 (proteins with at least two peptides are identified) -y 1 (partial tryptic end is allowed) --pfp 0.01 (protein FDR < 1%) -DM 5 (highest precursor mass error 5 ppm) -U (unique peptide only). Quantification was performed by Census [66] on IP2.

### Differential protein expression analysis

Proteomics data was first summarized to peptide level by adding up the intensities of constituting spectra. Quantitation results from different TMT runs were merged and normalized using the pooled samples channel which was present in all runs. For each peptide, multiple measurements from the same mouse were collapsed to one measurement by taking the sum of all measurements. Batch effects from the summarized protein-level data for each dataset were removed using ComBat [67]. The data were then log_2_ transformed. Differentially expressed proteins were identified using function lmFit in limma [68] followed by eBayes moderation of standard errors [69]. Resulting *P*-values were FDR-corrected using the Benjamini–Hochberg method to control for multiple comparisons.

### Weighted protein co-expression network analysis

We used weighted protein co-expression network analysis (WPCNA) [70] to define modules of co-expressed proteins from proteomics data. Proteomics data was first summarized to protein level by adding up the channel intensities of constituting peptides for each of six datasets derived from two brain regions (cortex and cerebellum) and three developmental periods (embryonic, early postnatal and adult) (Supplementary Fig. S11). Batch effects from the summarized protein-level data for each dataset were removed using ComBat [67], followed by log_2_ transformation. Modules were estimated using the blockwiseModules function with the following parameters: corType = bicorr; networkType = signed; pamRespectsDendro = F; mergeCutHeight = 0.1. Some parameters were specific to each dataset. For embryonic cortex: power = 18; deepSplit = 0; minModuleSize = 100; for early postnatal cortex: power = 18; deepSplit = 0; minModuleSize = 100; for adult cortex: power = 18; deepSplit = 0; minModuleSize = 20; for embryonic cerebellum: power = 22; deepSplit = 0; minModuleSize = 40; for early postnatal cerebellum: power = 26; deepSplit = 0; minModuleSize = 150; and for adult cortex: power = 12; deepSplit = 2; minModuleSize = 40. Module eigengene-genotype associations were calculated using linear regression model. Significance *P*-values were FDR-corrected to account for multiple comparisons. Genes within each module were prioritized based on their module membership (kME), defined as correlation to the module eigengene.

### Transcriptome vs proteome correlation

Correlation studies between transcriptomes and proteomes for cortex and cerebellum were performed in R using cor.test function. To obtain the datasets, DEGs for each brain region and corresponding developmental period were intersected with the corresponding proteomes, and the expression log fold changes of both DEGs and corresponding proteins were used to calculate Pearson correlation coefficients (*R*) and *P*-values. *R* were also calculated for each brain region by combining DEGs/proteins from different periods to increase power.

### *Cul3* expression in human and mouse published datasets

Human RNA-seq data (as RPKM) for neocortex was downloaded from the BrainSpan Atlas of the Developing Human Brain (https://hbatlas.org/hbtd/basicSearch.pl). For the developmental trajectory, Neocortex (NCX) was selected and the expression of Cul3 was plotted for time points up to 37 years of age. The data were plotted as mean across samples, error bar represents standard deviation. For expression profile of Cul3 in mouse, the data from C57Bl6 mice were extracted from Gompers et al. [71] and Cul3 expression was plotted starting from E12.5 up to adult.

### Cell type enrichment analysis using mouse scRNA-seq data

Cell-type enrichment analysis for each protein co-expression module was performed using the expression weighted cell type enrichment (EWCE) package in R [72]. Cell type-specific gene expression data was obtained from single cell sequencing (scRNA-seq) studies of Postnatal (P0) mouse neocortex regions [24]. The specificity metric of each gene for each cell type was computed as described [72]. “Intermediate progenitors” cell type includes a union of SVZ 2 (migratory neurons), “Interneurons” cell type includes union of Interneuron 5P, 6P, 11P, and 14P, “Layer V–VI” cell type includes 18P and 12P (excitatory neurons), and “Layer II–IV” cell type includes 15P and 1P (excitatory neurons). Enrichment was evaluated using bootstrapping. *Z*-score was estimated by the distance of the mean expression of the target gene set from the mean expression of bootstrapping replicates. *P*-values were corrected for multiple comparisons using FDR.

### Western blotting

The small fraction of tissue lysates prepared for mass-spectrometry experiments were used for western blotting. On average 10–15 μg of total protein from WT and *Cul3*^+/−^ cerebral cortex, cerebellum or hippocampus were resolved by SDS-PAGE and transferred onto PVDF Immobilon-P membranes (Millipore). After blocking with 5% nonfat dry milk in 1× TBS with 0.1% Tween-20 (TBST) for 1 h at room temperature, membranes were first probed overnight with the appropriate primary antibodies in 3% BSA in TBST, and then after 1 h of incubation with corresponding host specific HRP-conjugated secondary antibody (Abcam). Membranes were developed using the EZ-ECL chemiluminescence detection kit (Denville Scientific). The following primary antibodies were used: anti-Cul3 (1:1000; Cell Signaling), anti-RhoA (1:1000; Cell Signaling), and anti-Gapdh (1:5000; Sigma Aldrich) as a loading control. Quantification was performed by densitometry with ImageJ software. Western Blot images for quantification of Cul3, total RhoA and active RhoA are shown in the Supplementary Fig. S18.

### Quantification and statistical analysis

The statistical analyses for the above experiments were performed using Prism software (GraphPad). Statistical tests, sample sizes, and exact *P*-values are described in Figure legends. Significance was defined as *p* < 0.05(*), *p* < 0.01(**), or *p* < 0.001(***). Blinded measurements were performed for any comparison between control and *Cul3*^+/−^ genotypes.

## Supplementary information

Supplementary table S1

Supplementary table S2

Supplementary table S3

Supplementary table S4

Supplementary table S5

Supplementary table S6

Supplementary table S7

Supplementary table S8

Supplementary table S9

Supplementary table S10

Supplementary table S11

Supplementary table S12

Supplementary table S13

Supplementary table S14

Supplementary table S15

Supplementary Figure 1

Supplementary Figure 2

Supplementary Figure 3

Supplementary Figure 4

Supplementary Figure 5

Supplementary Figure 6

Supplementary Figure 7

Supplementary Figure 8

Supplementary Figure 9

Supplementary Figure 10

Supplementary Figure 11

Supplementary Figure 12

Supplementary Figure 13

Supplementary Figure 14

Supplementary Figure 15

Supplementary Figure 16

Supplementary Figure 17

Supplementary table S16

Supplementary Figure 18

Supplementary Figures legends

## Data Availability

Source RNA-seq data is available at GEO repository accession number GSE144046. Source proteomics data is available from the public repository MassIVE (Mass Spectrometry Interactive Virtual Environment), a part of the ProteomeXchange consortium, with the identifier MSV000084830 (and PXD017256 for ProteomeXchange) through the following link (https://massive.ucsd.edu/ProteoSAFe/dataset.jsp?accession=MSV000084830).

## Code availability

The code used for the RNAseq and proteomics analysis generated during this study is available from GitHub (https://github.com/IakouchevaLab/CUL3).
