# Supplementary figures and images for: Autism-linked Cullin3 germline haploinsufficiency impacts cytoskeletal dynamics and cortical neurogenesis through RhoA signaling
Source: Mol Psychiatry. 2021 Mar 16;26(7):3586–613. doi: 10.1038/s41380-021-01052-x (PMC8443683; doi:10.1038/s41380-021-01052-x)

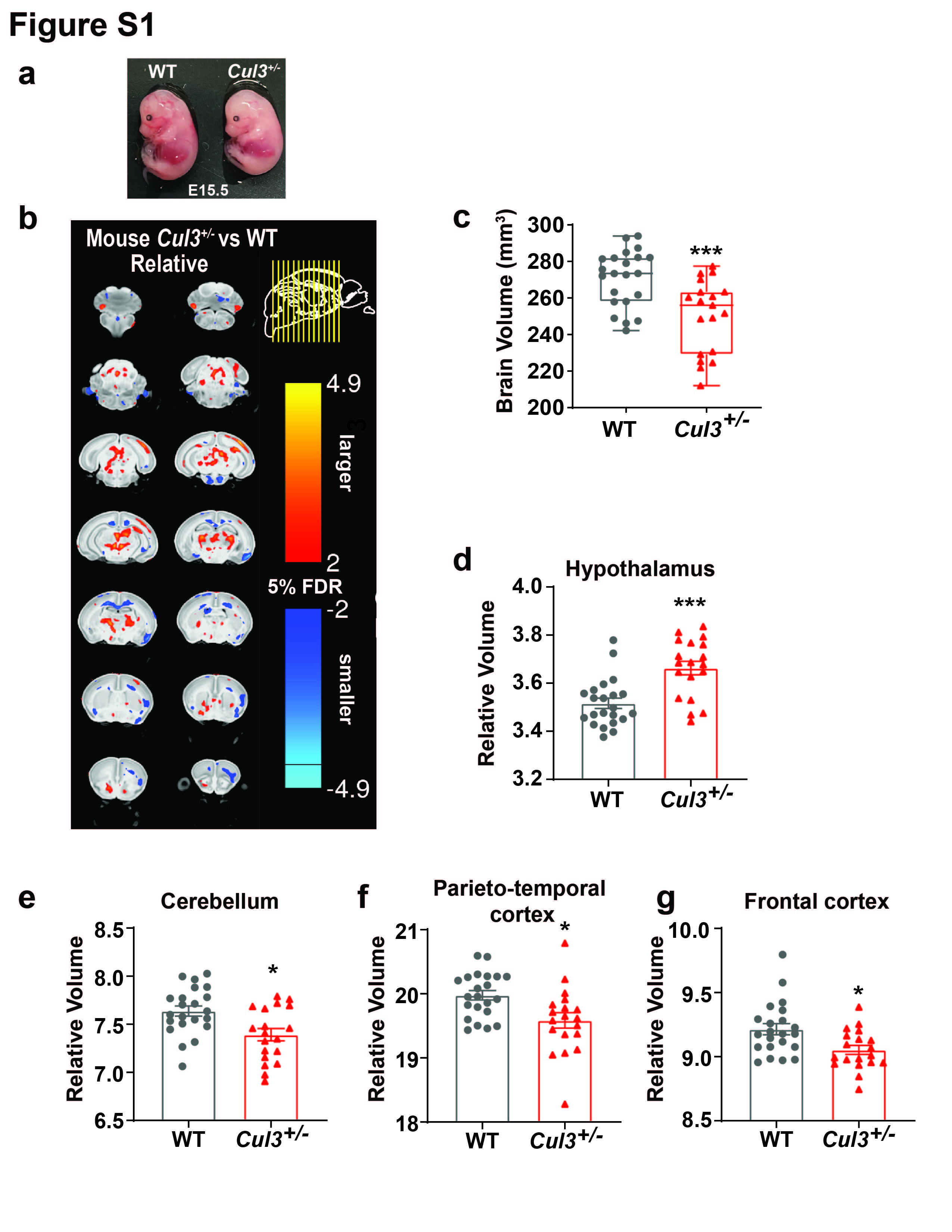

Supplement: Supplementary file 16 — Supplementary Figure 1 [file 41380_2021_1052_MOESM16_ESM.jpg]

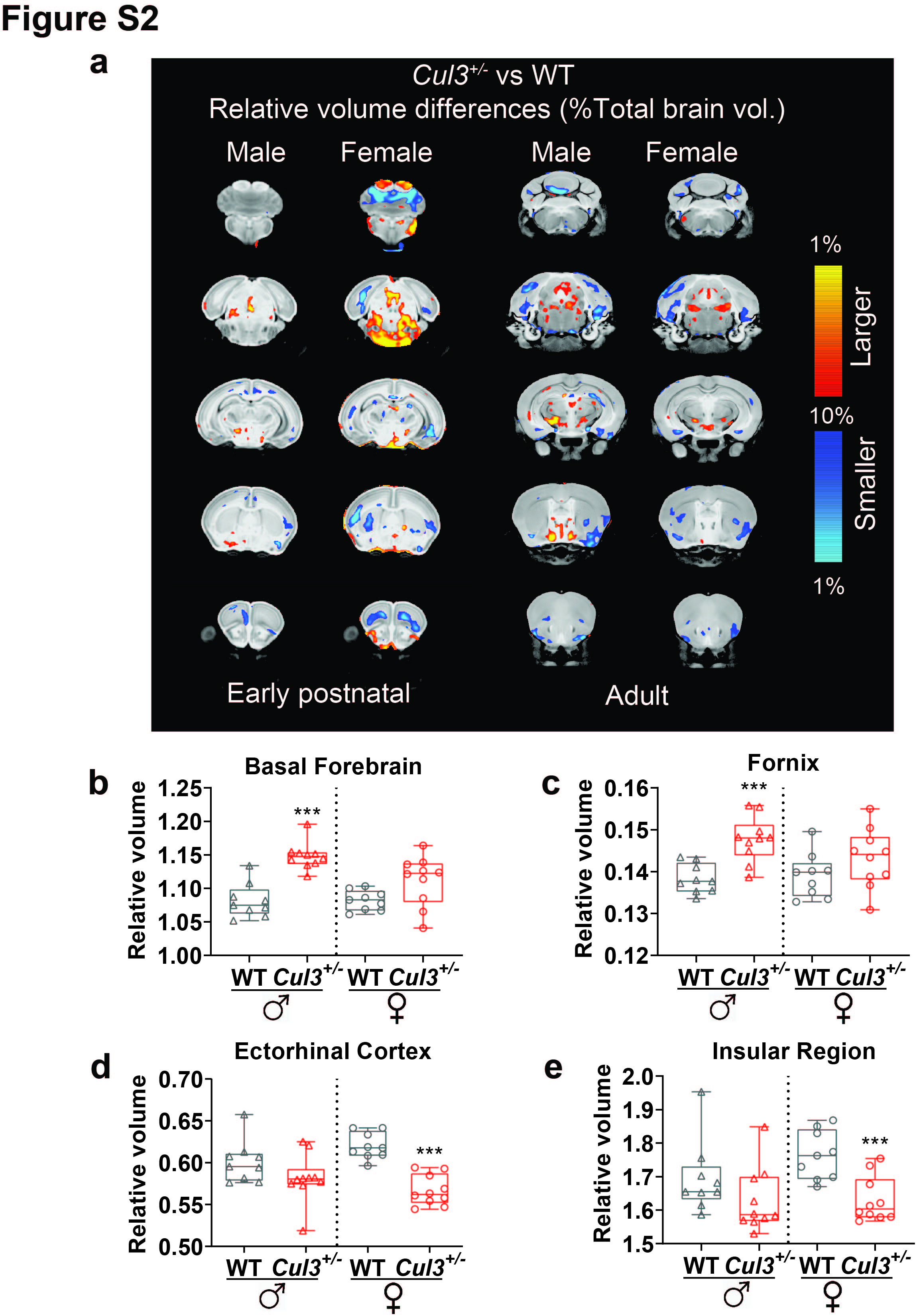

Supplement: Supplementary file 17 — Supplementary Figure 2 [file 41380_2021_1052_MOESM17_ESM.jpg]

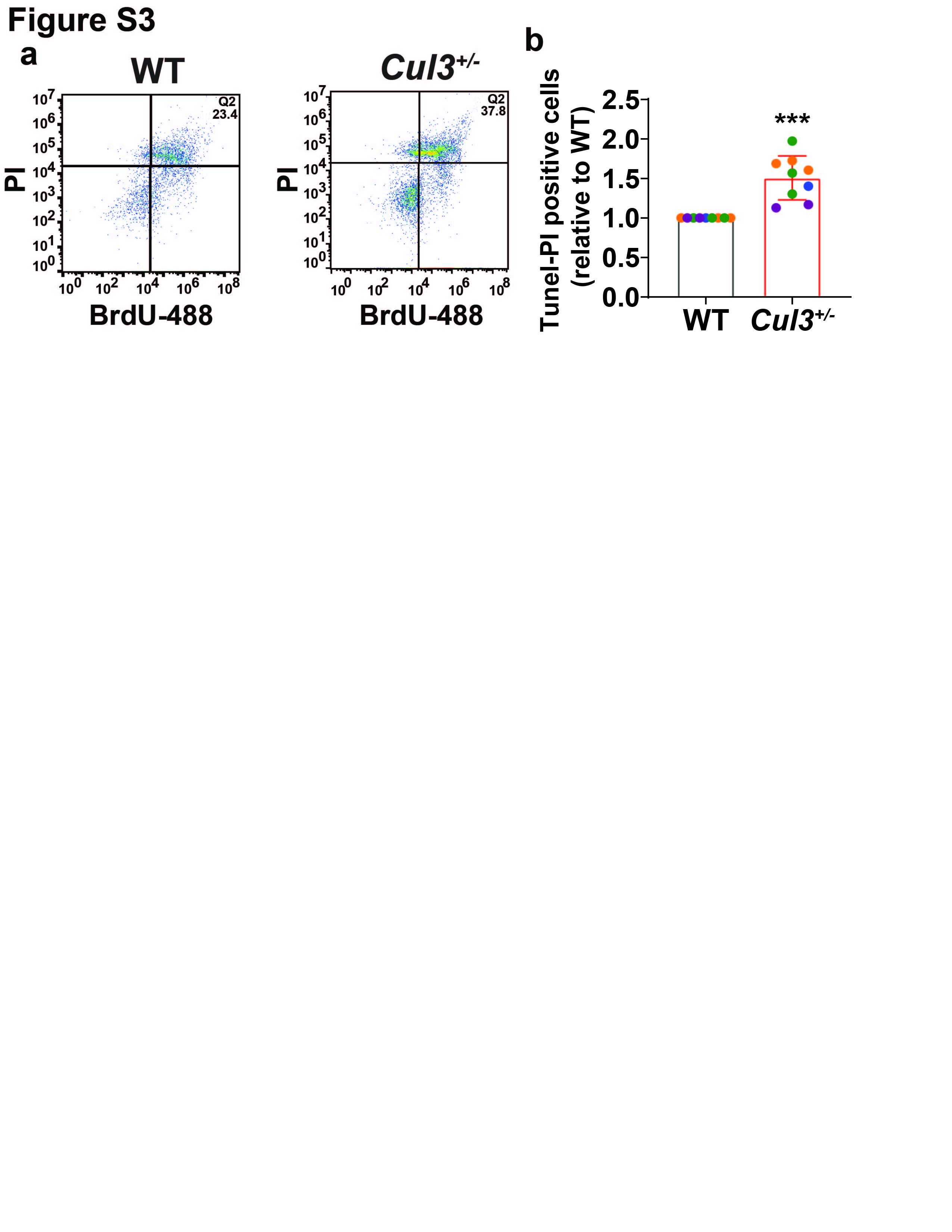

Supplement: Supplementary file 18 — Supplementary Figure 3 [file 41380_2021_1052_MOESM18_ESM.jpg]

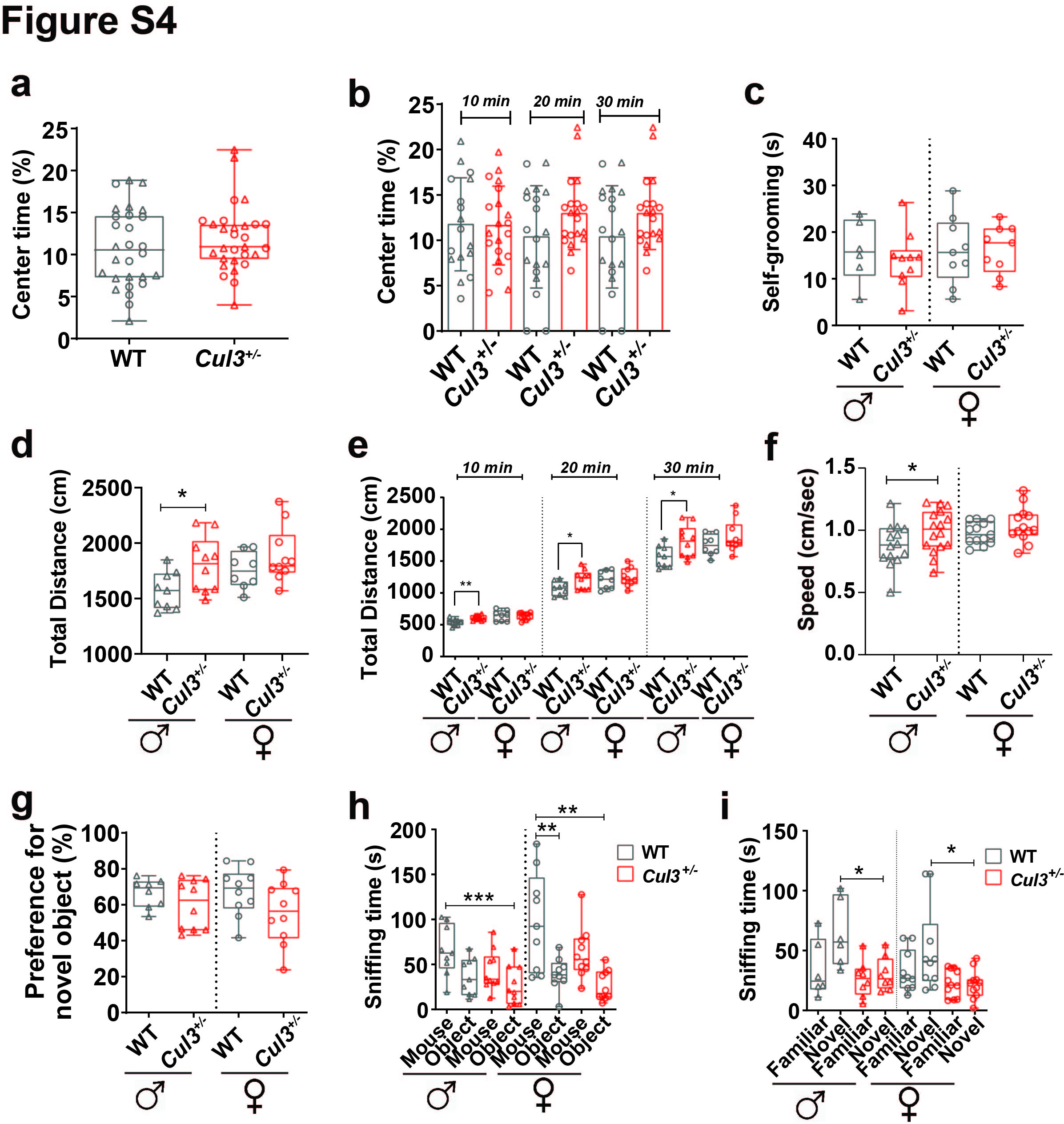

Supplement: Supplementary file 19 — Supplementary Figure 4 [file 41380_2021_1052_MOESM19_ESM.jpg]

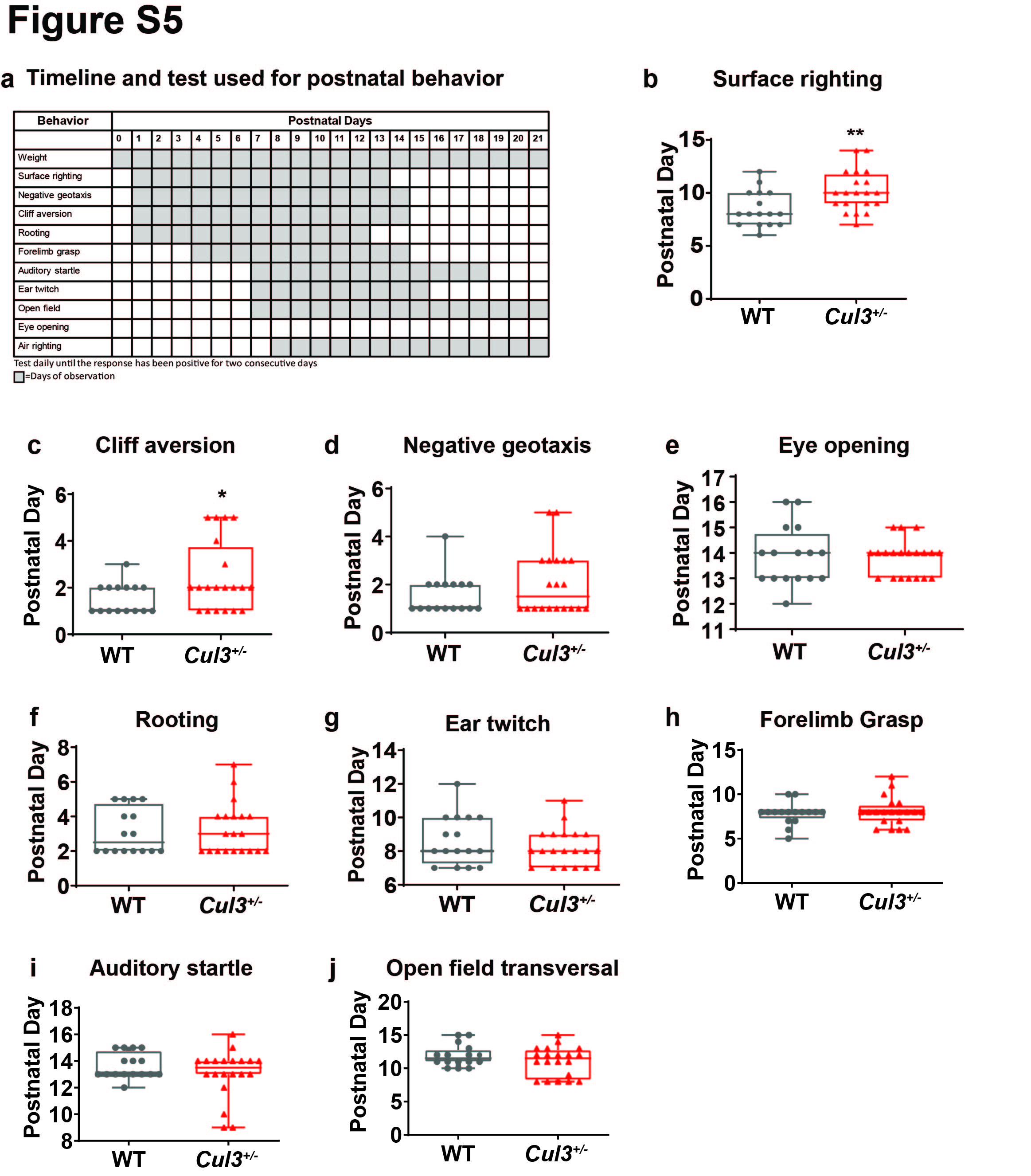

Supplement: Supplementary file 20 — Supplementary Figure 5 [file 41380_2021_1052_MOESM20_ESM.jpg]

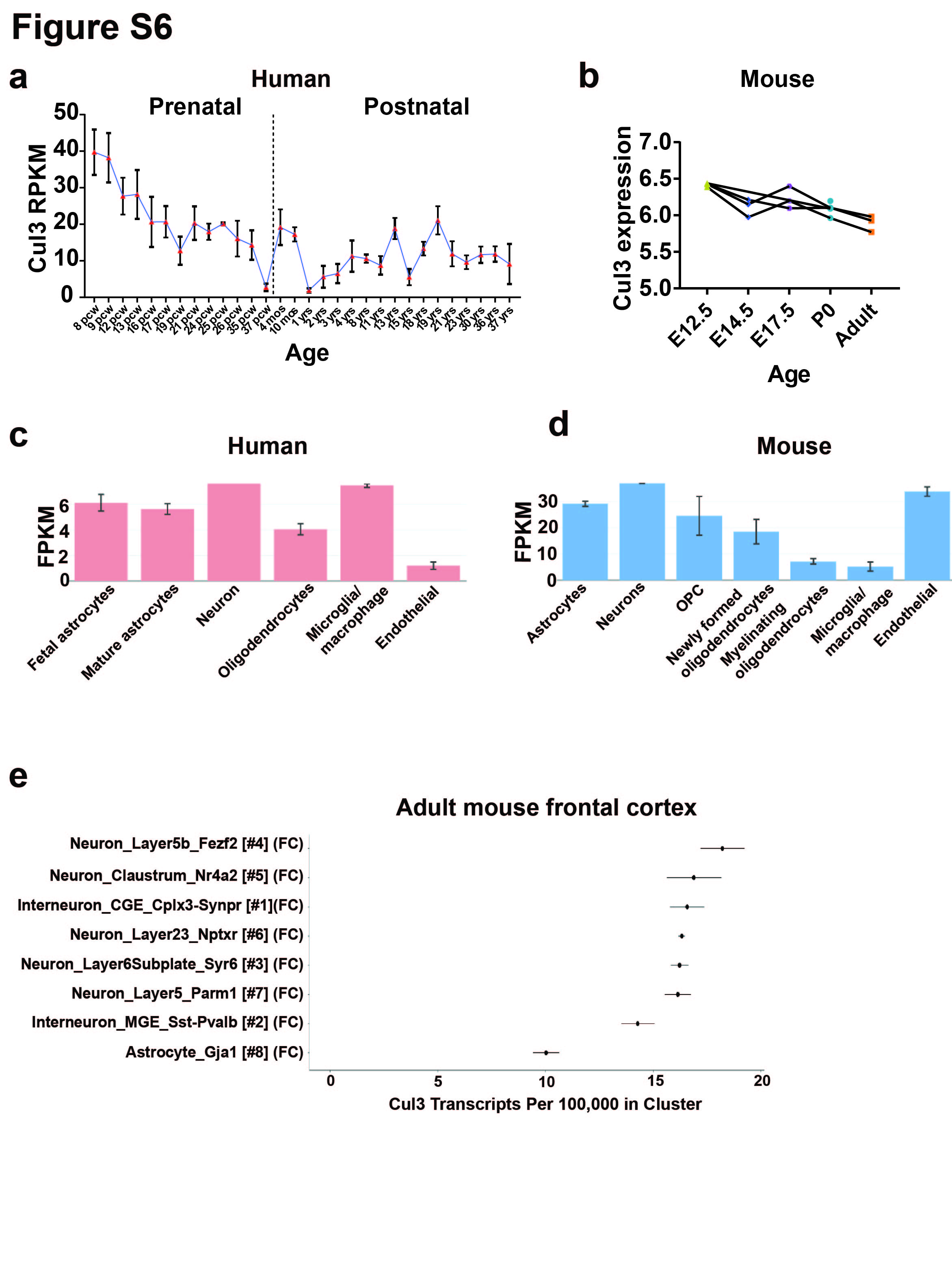

Supplement: Supplementary file 21 — Supplementary Figure 6 [file 41380_2021_1052_MOESM21_ESM.jpg]

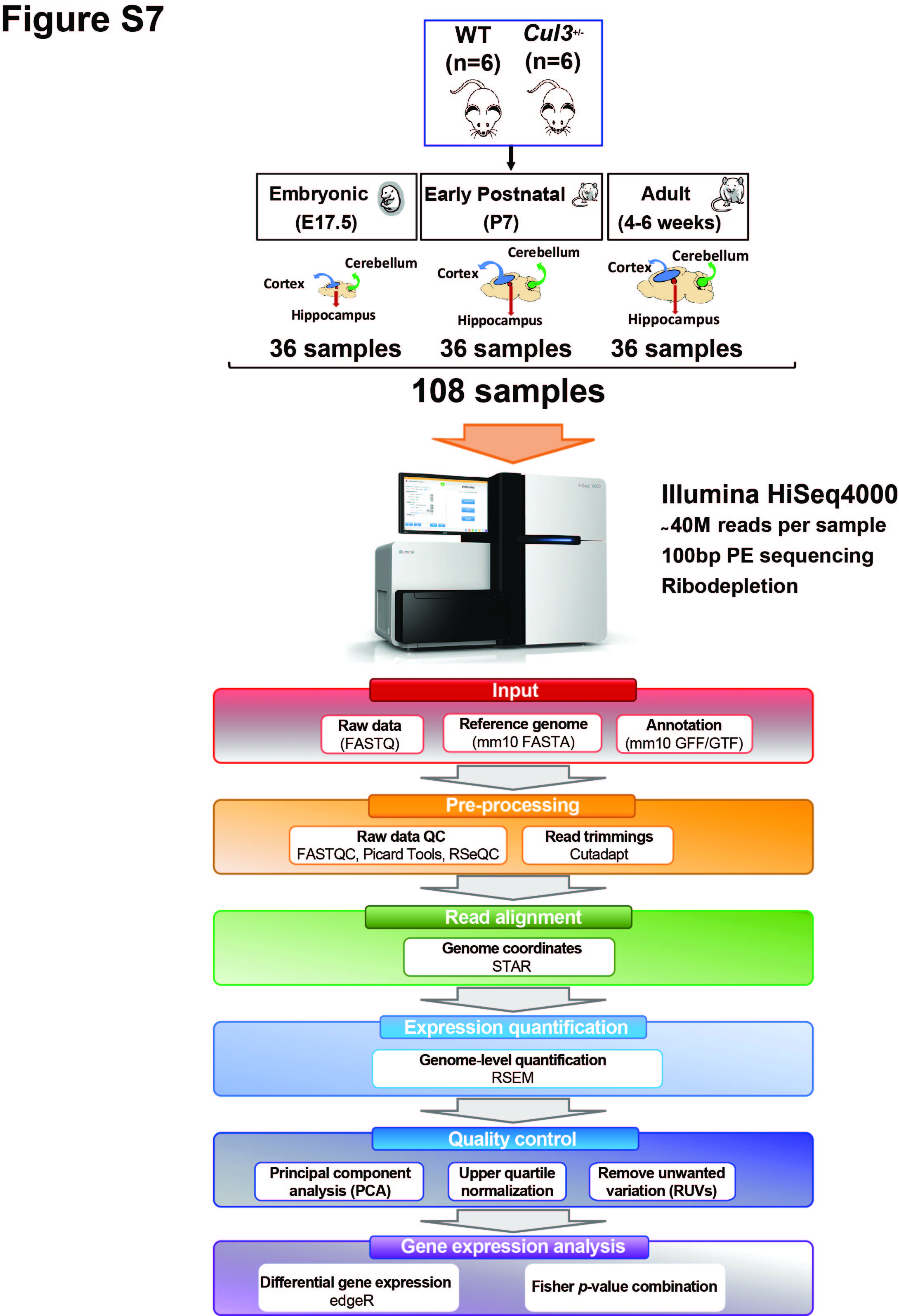

Supplement: Supplementary file 22 — Supplementary Figure 7 [file 41380_2021_1052_MOESM22_ESM.jpg]

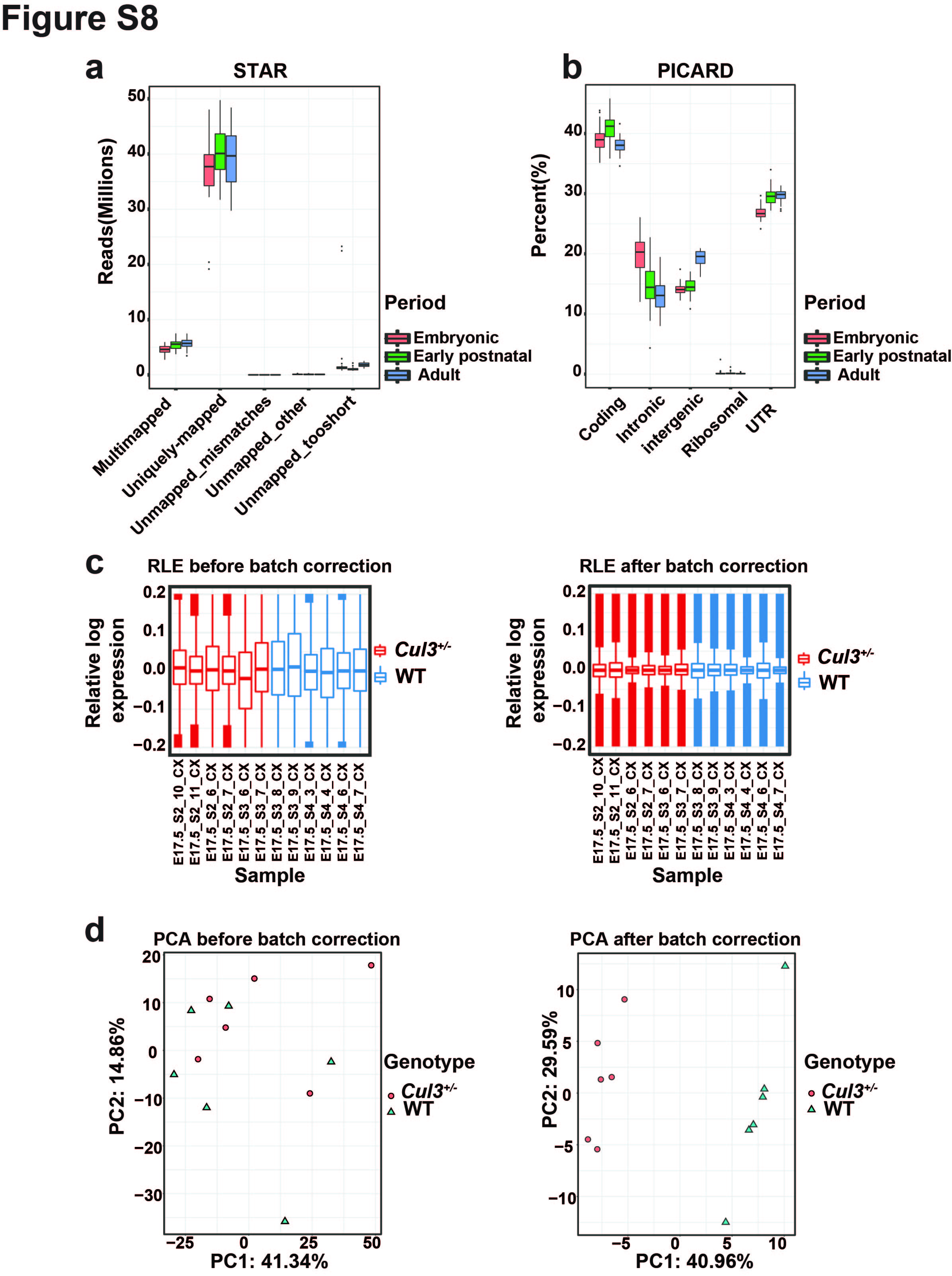

Supplement: Supplementary file 23 — Supplementary Figure 8 [file 41380_2021_1052_MOESM23_ESM.jpg]

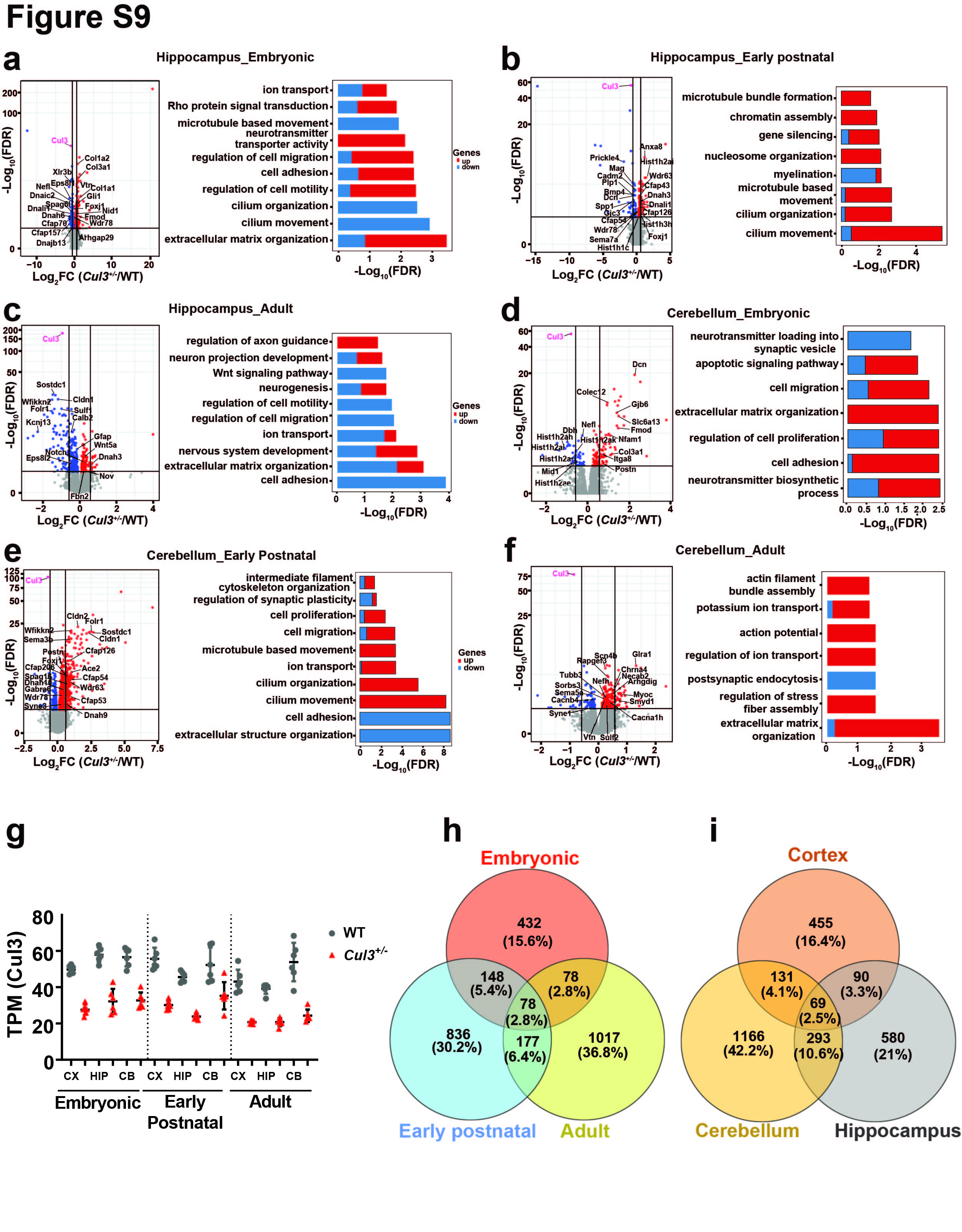

Supplement: Supplementary file 24 — Supplementary Figure 9 [file 41380_2021_1052_MOESM24_ESM.jpg]

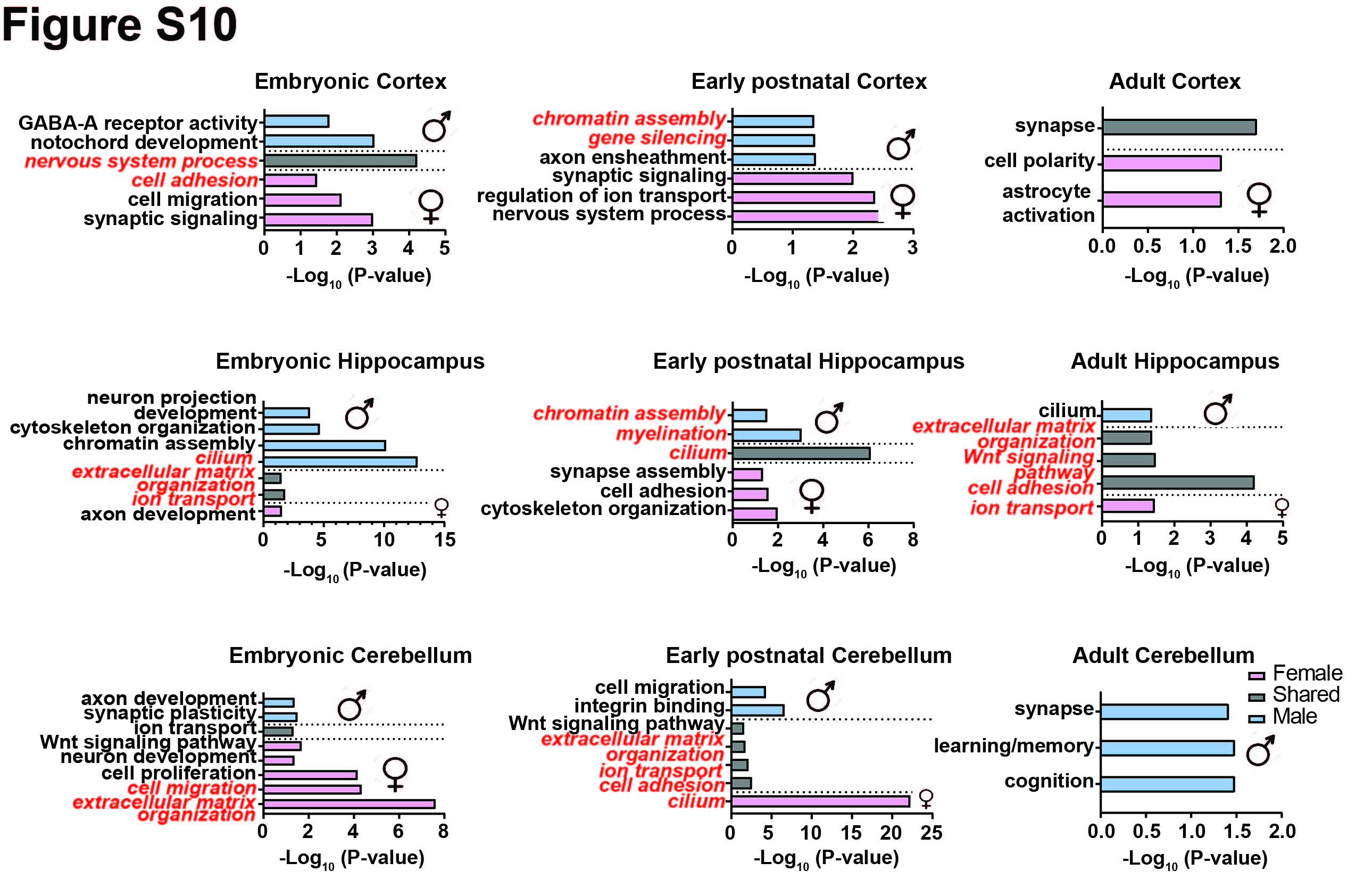

Supplement: Supplementary file 25 — Supplementary Figure 10 [file 41380_2021_1052_MOESM25_ESM.jpg]

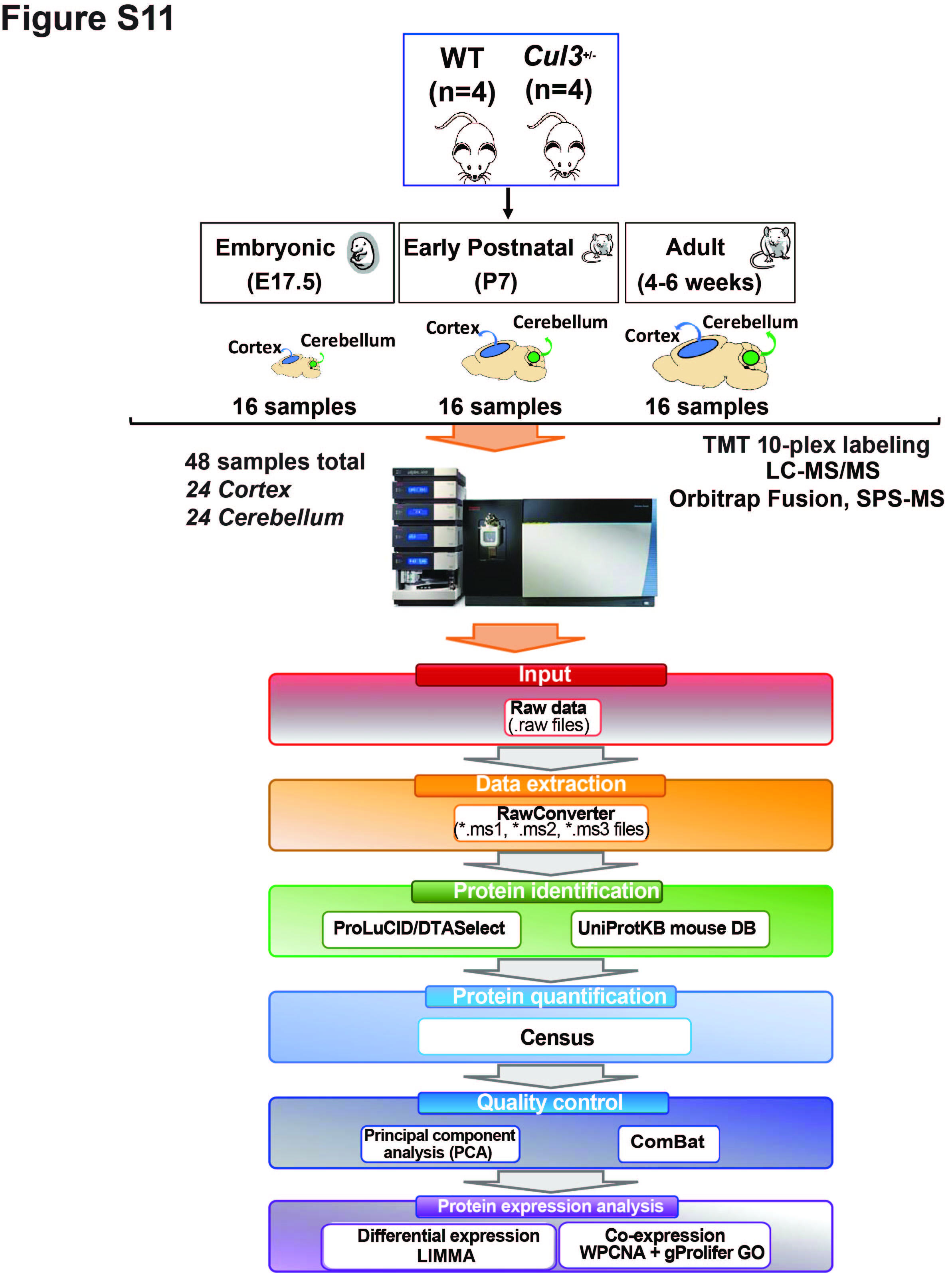

Supplement: Supplementary file 26 — Supplementary Figure 11 [file 41380_2021_1052_MOESM26_ESM.jpg]

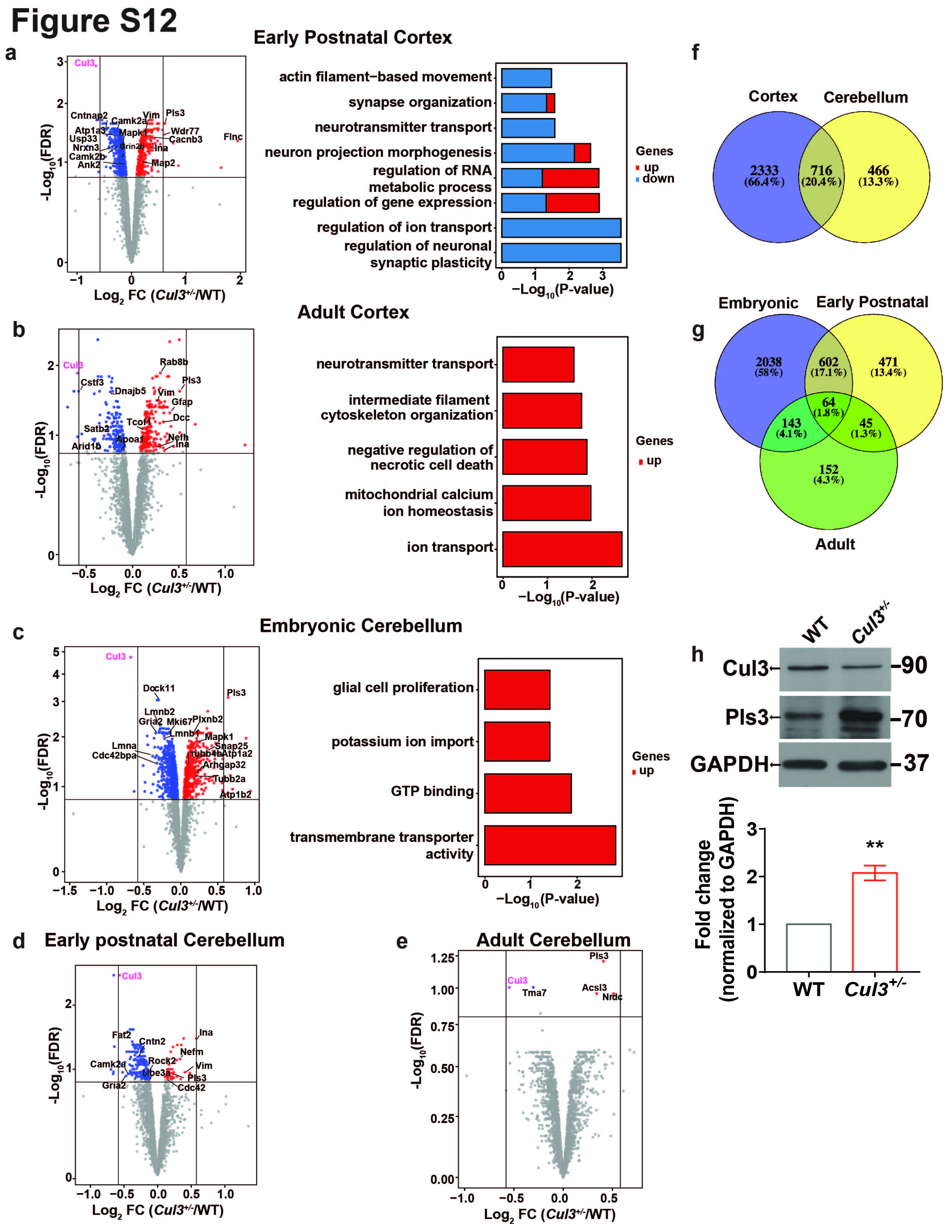

Supplement: Supplementary file 27 — Supplementary Figure 12 [file 41380_2021_1052_MOESM27_ESM.jpg]

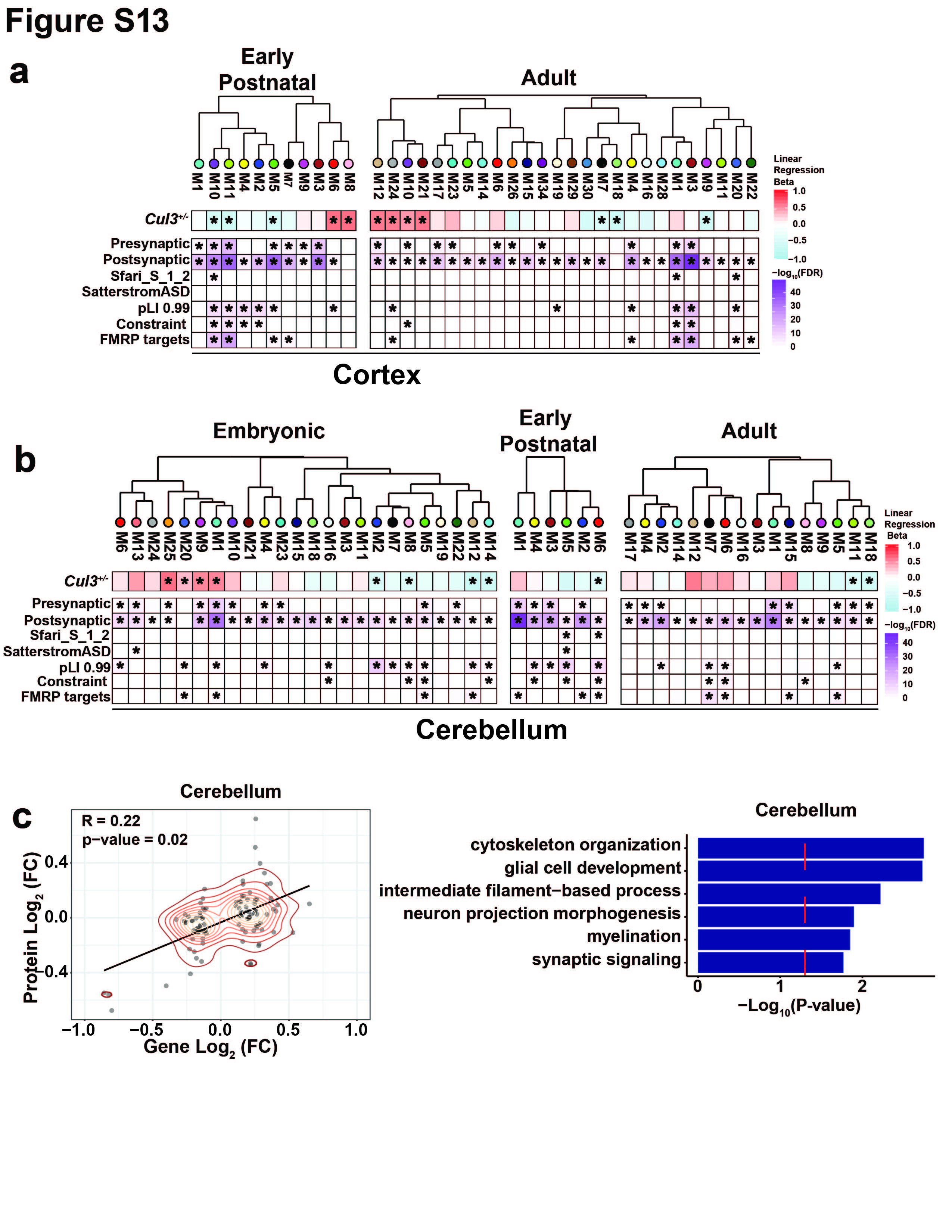

Supplement: Supplementary file 28 — Supplementary Figure 13 [file 41380_2021_1052_MOESM28_ESM.jpg]

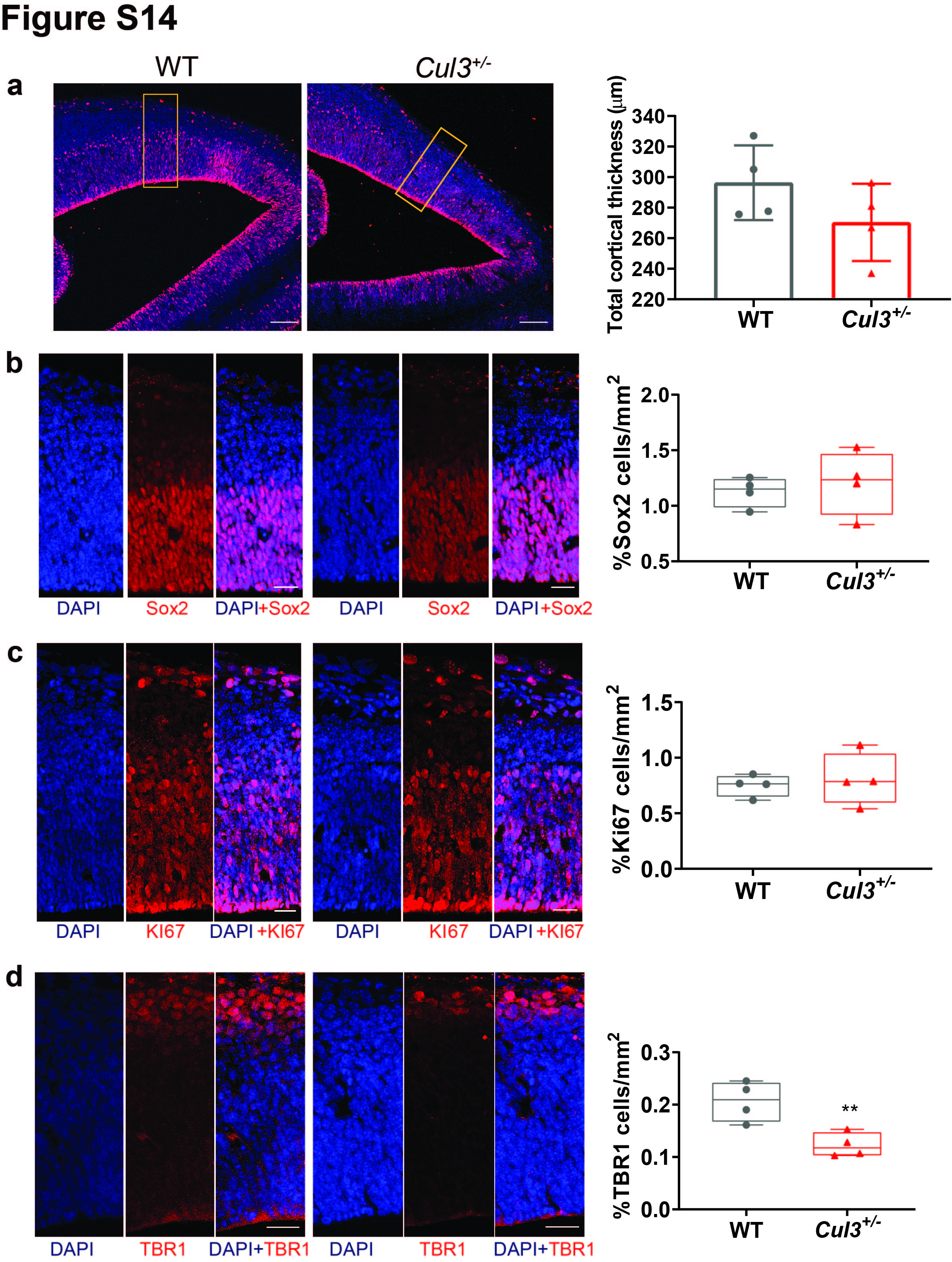

Supplement: Supplementary file 29 — Supplementary Figure 14 [file 41380_2021_1052_MOESM29_ESM.jpg]

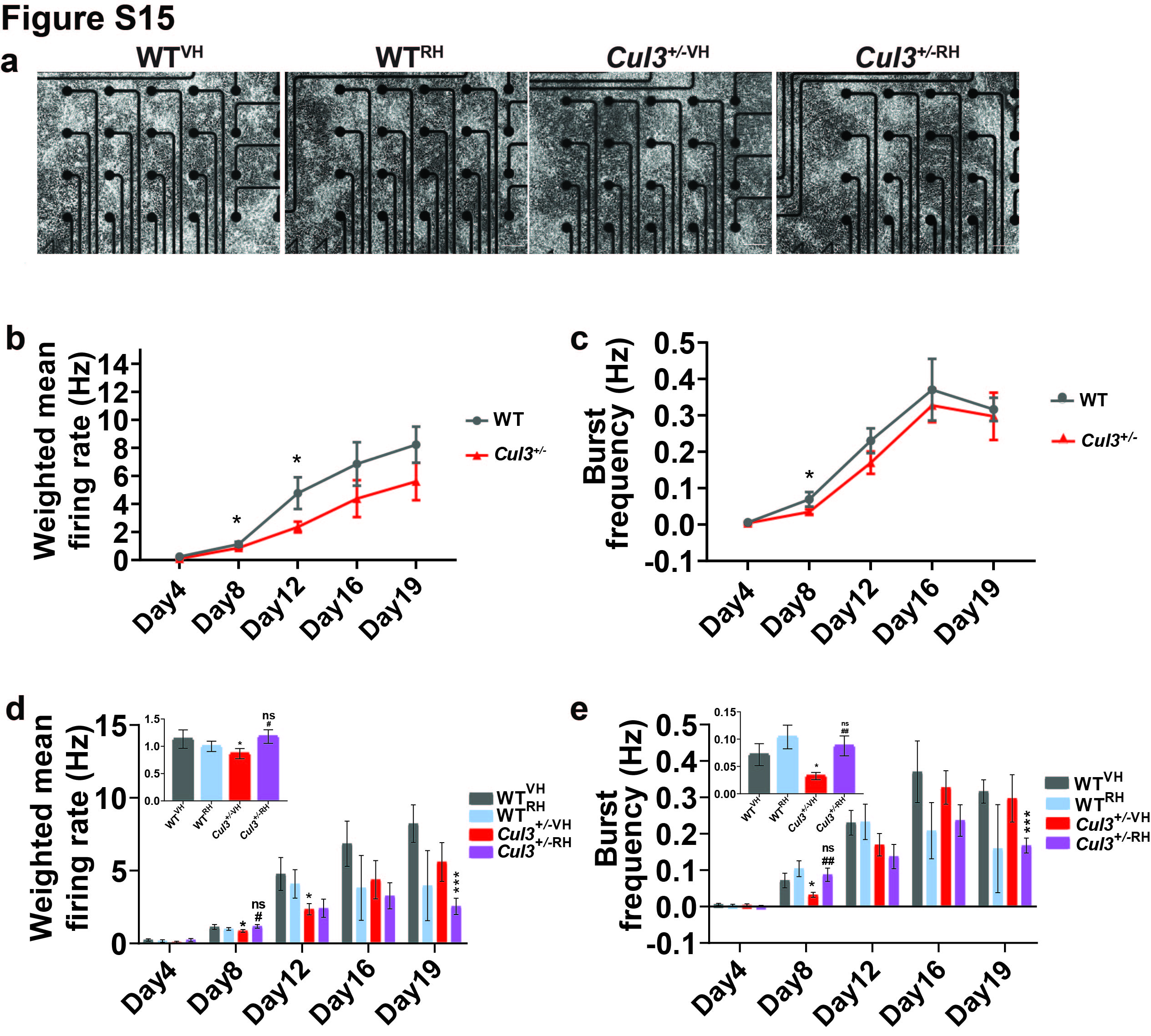

Supplement: Supplementary file 30 — Supplementary Figure 15 [file 41380_2021_1052_MOESM30_ESM.jpg]

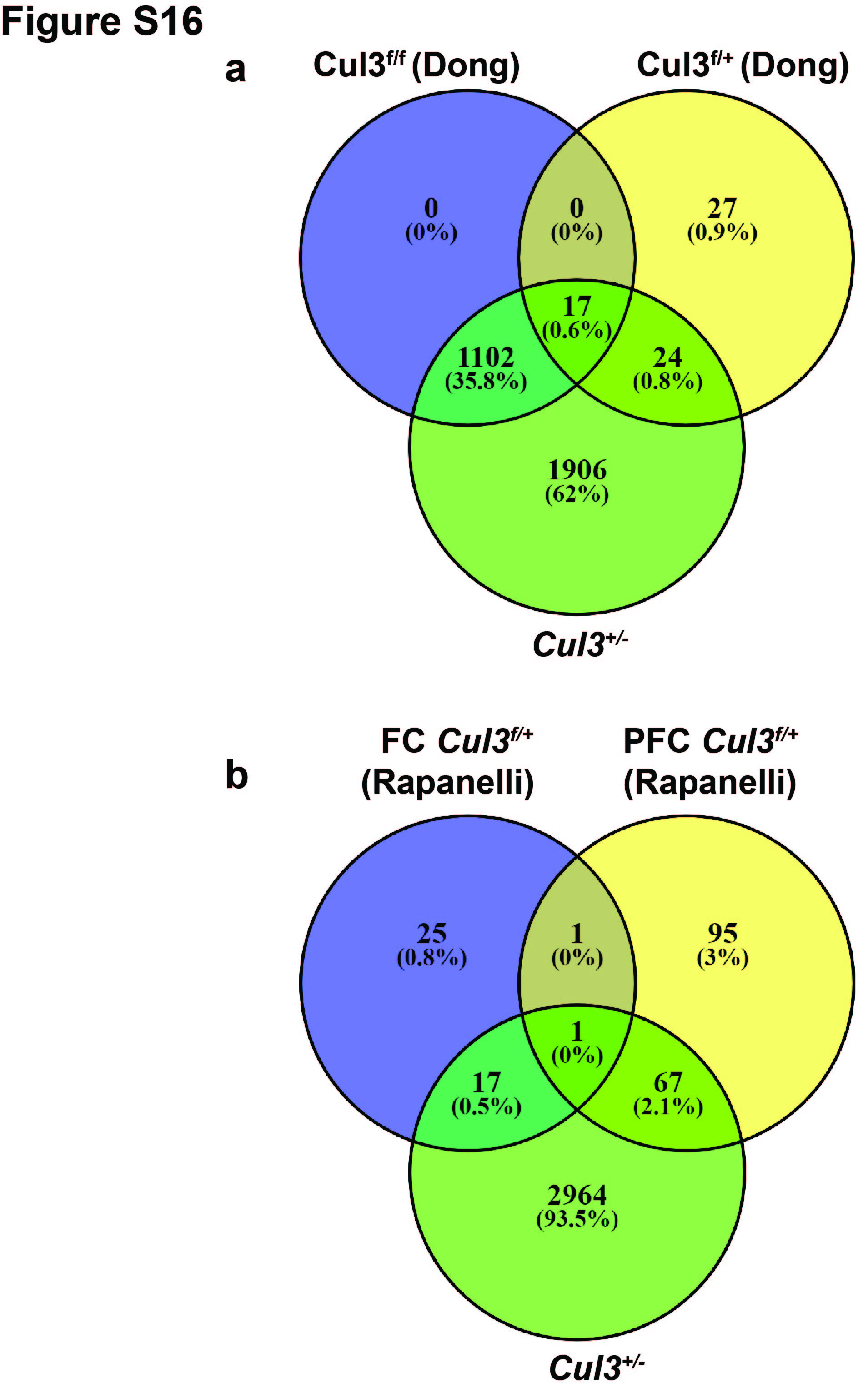

Supplement: Supplementary file 31 — Supplementary Figure 16 [file 41380_2021_1052_MOESM31_ESM.jpg]

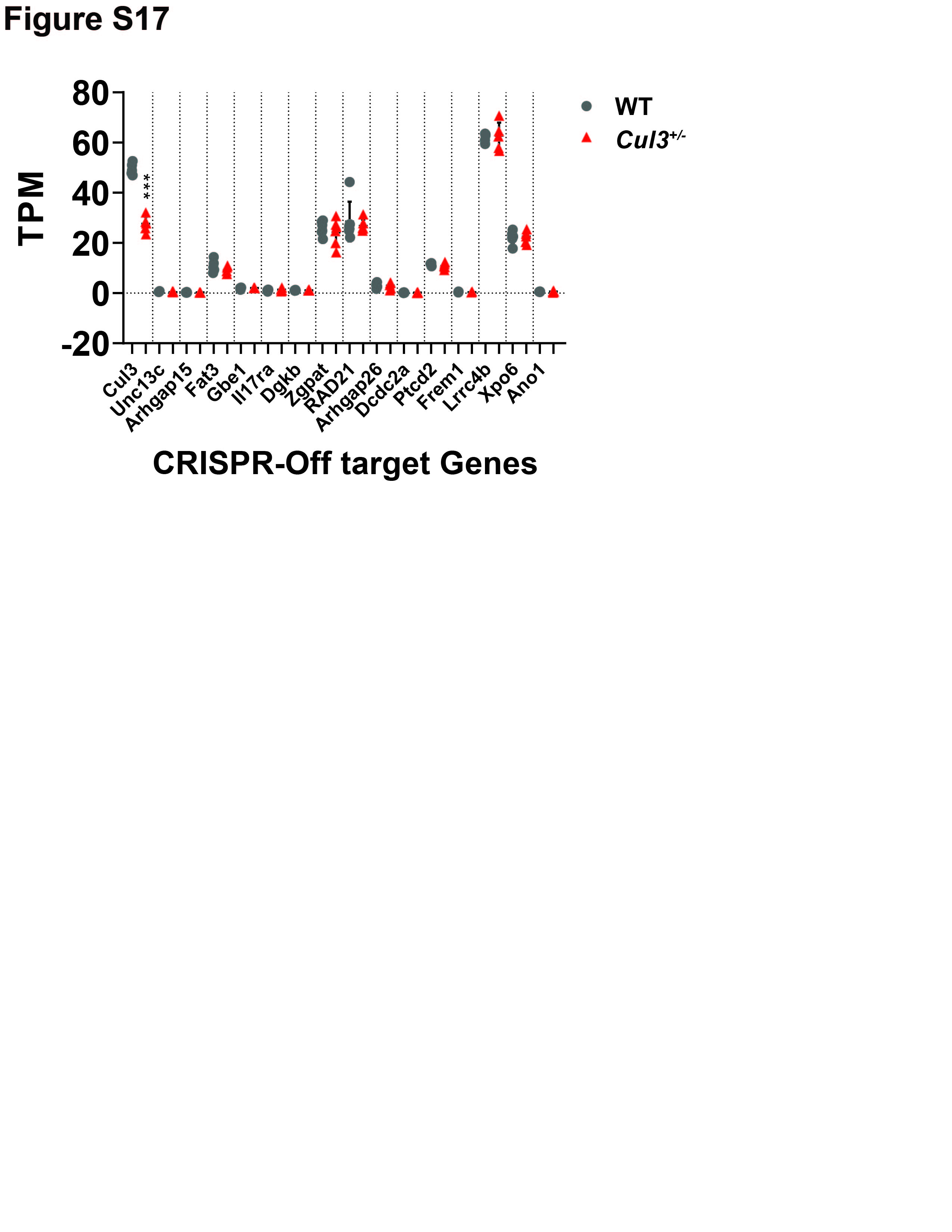

Supplement: Supplementary file 32 — Supplementary Figure 17 [file 41380_2021_1052_MOESM32_ESM.jpg]

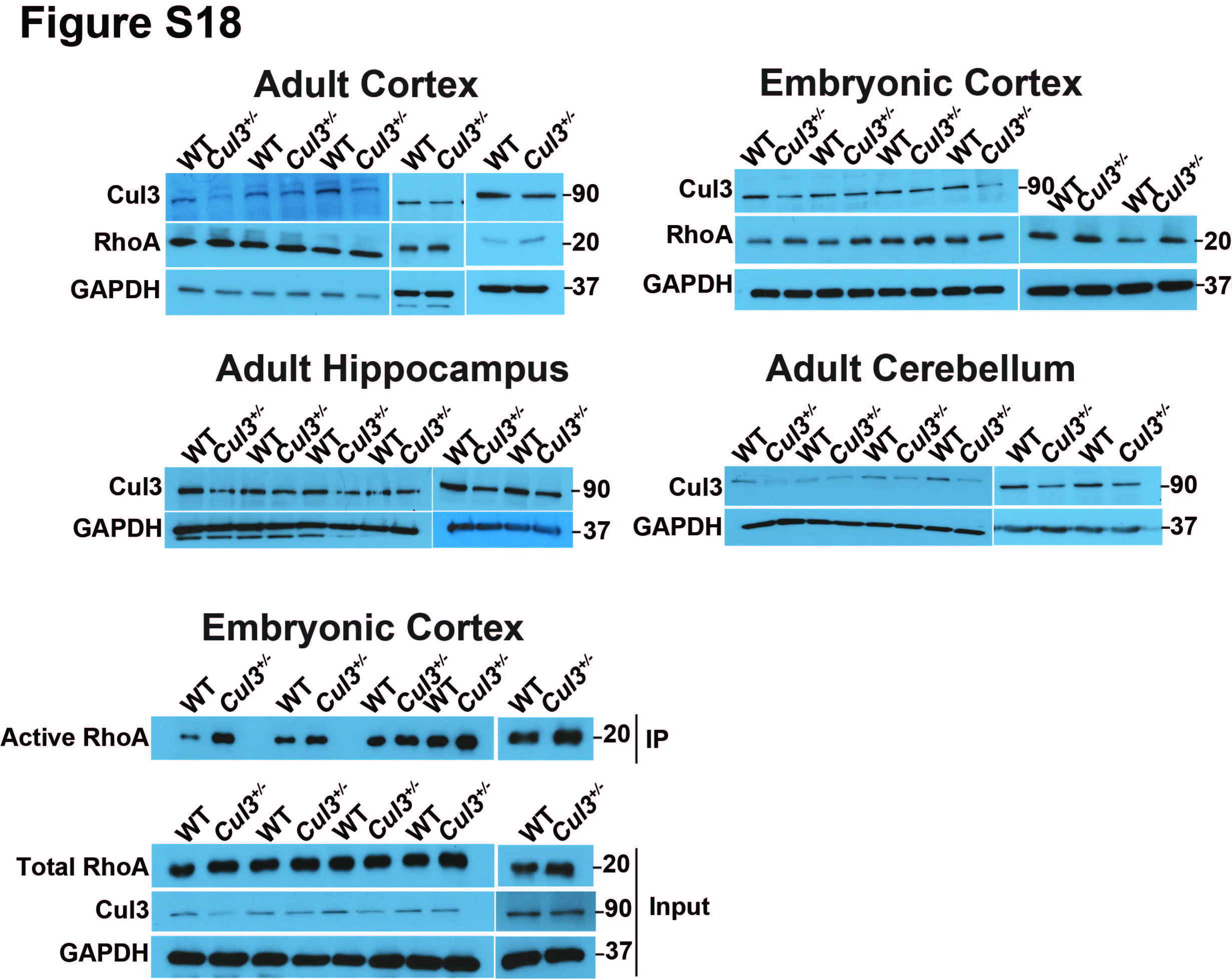

Supplement: Supplementary file 34 — Supplementary Figure 18 [file 41380_2021_1052_MOESM34_ESM.jpg]
