# Supplementary material for: Autism-linked Cullin3 germline haploinsufficiency impacts cytoskeletal dynamics and cortical neurogenesis through RhoA signaling
Source: Mol Psychiatry. 2021 Mar 16;26(7):3586–613. doi: 10.1038/s41380-021-01052-x (PMC8443683; doi:10.1038/s41380-021-01052-x)
Supplement: Supplementary file 35 — Supplementary Figures legends [file 41380_2021_1052_MOESM35_ESM.docx]

Supplementary Figures legends

**Figure S1. Altered brain morphology of *Cul3^+/-^* postnatal day 7 (P7) mice**. **(a)** The representative images of WT and *Cul3^+/-^* E15.5 embryos, *Cul3^+/-^* embryos are smaller in size. **(b)** Voxel-wise analysis highlighting significant differences in relative volumes throughout the brain between WT and *Cul3^+/-^* mice with 5% false discovery rate (FDR). Scale bar 2-4.9 indicates decreasing FDR, where the value of 2 corresponds to 5% FDR, and positive or negative values are *vs* WT brain. **(c)** *Cul3^+/-^* mice have reduced absolute brain volume (***p<0.001) compared with WT mice (n=22 WT, 19 *Cul3^+/-^*). **(d)** MRI revealed significant increase in hypothalamus volume (****p*<0.001). (**e-g**) MRI revealed significant reduction in relative volume (normalized by % total brain volume) of cerebellum (*p<0.05), parieto-temporal cortex (*p<0.05), and frontal cortex (*p<0.05); Dots represent individual samples; two tailed t-test was used for **c-g**; error bars represent mean ± SD.

**Figure S2. Altered brain morphology of male and female *Cul3^+/-^* postnatal and adult mice. (a)** Voxel-wise analysis highlighting significant differences in relative volumes throughout the brain between WT and *Cul3^+/-^* postnatal day 7 and adult male and female mice with 10% false discovery rate (FDR) and positive or negative values are *vs* WT brain. **(b-c)** *Cul3^+/-^* adult males have increased relative volume of basal forebrain and fornix (***p<0.001) compared with WT mice (n=10 WT, N=9 *Cul3^+/-^*). **(d-e)** *Cul3^+/-^* adult females have reduced relative volume of ectorhinal cortex and insular region (***p<0.001) compared with WT mice (n=10 WT, N=9 *Cul3^+/-^*). Dots represent individual samples; two tailed t-test was used for **b-e**; error bars represent mean ± SD.

**Figure S3. Increased apoptosis in *Cul3^+/-^* mice.** **(a-b)** TUNEL assay on 14DIV primary cortical neurons, the dual positive neurons for PI and BrdU-488 were counted by flow cytometry; (Q4) were considered as apoptotic cells containing BrdU-488/PI+ cells (***p<0.001; n=9 for each genotype). Dots represent independent samples; two tailed t-test used for **b**; error bars represent mean ± SD

**Figure S4**. ***Cul3^+/-^* mouse behavioral phenotypes including the analyses by sex. (a)** *Cul3^+/-^* mice display no preference for the center *vs* periphery in open field test. **(b)** 10 min time-bins showing no difference in % time spent in the center in any 10 min intervals. **(c)** No difference in time spent in self-grooming between WT and *Cul3^+/-^* mice (WT n=6-9/sex; *Cul3^+/-^* n=9-10/sex). **(d)** *Cul3^+/-^* males travel longer distances in open field (n=9/sex WT; n=10/sex *Cul3^+/-^* (*p<0.05) compared with WT mice. **(e)** The travelling speed (cm/sec) is significantly increased in *Cul3^+/-^* male mice (*p<0.05). **(f)** Time bins showing that *Cul3^+/-^* male mice travel significantly longer distances in each 10 min bin of the test (* p<0.05; **p<0.01). **(g)** *Cul3^+/-^* male and female mice demonstrate no difference in preference for novel object in novel object recognition test (WT n=8/sex; *Cul3^+/-^* n=10/sex). **(h)** Insignificant reduction in sniffing time was observed for *Cul3^+/-^* male and female mice while interacting with a novel mouse (*vs* novel object) as compared to WT mice (WT n=9/sex; *Cul3^+/-^* n=10/sex; **p<0.01; *p<0.05; One-way ANOVA). **(i)** Reduced sniffing time is observed for *Cul3^+/-^* male and female mice while interacting with a novel mouse (*vs* familiar mouse) as compared to WT mice (WT n=9/sex; *Cul3^+/-^* n=8-10/sex) *p<0.05; two tailed t-test for panels **a-g**; One- way ANOVA for **h-i**. Dots represent individual animals; ○-female and ∆-male; error bars represent mean ± SD.

**Figure S5. Analysis of developmental milestones and early postnatal behaviors in *Cul3^+/-^* mice. (a)** The timeline used for the early postnatal behavioral assays. **(b)** The righting reflex is motor ability for a mouse pup to be able to flip onto its feet from a supine position, *Cul3^+/-^* mice display delayed righting reflex as compared to WT mice (n=17 WT, n=19 *Cul3^+/-^,* p<0.01). **(c)** *Cul3^+/-^* mice display significant vestibular imbalance. The pup's eyes are still closed so fear is not the driving factor to turn away from the cliff's edge (n=15 WT, n=20 *Cul3^+/-^**, p<0.05). **(d-k)** Both *Cul3^+/-^* and WT mice have no difference in other postnatal behaviors tasks. Dots represent independent animals; two tailed t-test used for **b-k**; error bars represent mean ± SD.

**Figure S6. Cul3 expression in human and mouse across development using publicly available data**. **(a)** Cul3 expression in the developing human neocortex extracted from Brainspan (<https://www.brainspan.org/>). **(b)** Cul3 expression in the developing mouse brain based on Gompers datasets ^68^. **(c)** Cell-type specific expression of *Cul3* gene in the human brain (<https://www.brainrnaseq.org/>) ^13^. **(d)** Cell-type specific expression of *Cul3* gene in the mouse brain (<https://www.brainrnaseq.org/>) ^14^. **(e)** Cell-type specific expression of *Cul3* in the mouse brain based on DropViz ([http://dropviz.org)](about:blank) ^15^.

**Figure S7**. **RNA-seq experimental design and data analysis workflow.** A total of 108 transcriptomes (n=6 animals for each genotype, 3 brain regions, 3 developmental periods) have been bulk RNA sequenced in this study. A rigorous quality control including principal component analyses, upper quartile normalization, and removal of unwanted variables (RUV) was used for batch correction.

**Figure S8**. **Quality control metrics for RNA-seq data.** **(a)** Sequencing metrics from STAR (2.5.3a) for samples from three periods (embryonic E17.5, early postnatal P7 and adult). **(b)** Sequencing metrics from PICARD (v2.12) from three periods. **(c)** Relative log expression values are shown before (left panel) and after (right panel) batch correction using RUVseq for the embryonic cortex samples as an example. **(d)** First two principal components (PCs) of gene expression values calculated using “prcomp” function in R, are shown before (left panel) and after (right panel) batch correction using RUVseq for the embryonic cortex samples as an example.

**Figure S9.** **Differential gene expression for individual periods and regions. (a-f)** (Left) Volcano plots of differentially expressed genes in *Cul3^+/-^* *vs* WT for embryonic, early postnatal and adult hippocampus and cerebellum. Genes colored in red are upregulated in *Cul3^+/-^* compared to WT; genes colored in blue are downregulated in *Cul3^+/-^* compared to WT; Cul3 is colored in pink. (Right) GO-terms enrichment of differentially expressed genes by periods and regions. Contribution of up- or down-regulated genes to specific GO terms are shown in blue and red, respectively. **(g)** Dot plot of TPM values of *Cul3* gene in different brain regions and periods. Dots represent individual animals. (**h**) Venn diagram showing 78 common DEGs across the developmental periods. **(i)** Venn diagram showing 69 common DEGs across brain regions.

**Figure S10.** **Spatio-temporal differential gene expression analysis by sex.** GO term enrichment of the DEGs with 10% FDR from males and females. GO terms shared by both sexes are highlighted in grey, the terms enriched in females are in pink, and the terms enriched in males are in blue (n=3 males and 3 females for each genotype for each time period). GO terms marked in red italic font are shared between sex-specific and sex-blind analyses.

**Figure S11. TMT quantitative proteomics experimental design and data analysis workflow.** A total of 48 proteomes (n=4 animals for each genotype, 2 brain regions, 3 developmental periods) have been processed in this study by TMT 10-plex labeling followed by LC-MS/MS. Protein Quantification was carried out by Census. Quality control including principal component analyses, ComBat has been performed. LIMMA was implemented for differential protein expression analyses.

**Figure S12. Differential protein expression analyses for individual periods and regions. (a-e)** Volcano plots of differentially expressed proteins in *Cul3^+/-^* *vs* WT (left column) for early postnatal and adult cortex, and embryonic, early postnatal and adult cerebellum. Proteins colored in red are upregulated in *Cul3^+/-^* compared to WT; proteins colored in blue are downregulated in *Cul3^+/-^* compared to WT. (Right) GO-terms enrichment of differentially expressed proteins by regions. Contribution of up- or down-regulated proteins to specific GO terms are shown in blue and red, respectively. Early postnatal and adult cerebellum have no GO-term enrichment. **(f)** Venn diagram showing 716 shared DEPs across brain regions (FDR ≤ 0.15). **(g)** Venn diagram showing 64 shared DEPs across three developmental periods (FDR ≤ 0.15). **(h)** Western blot of Pls3 in embryonic cortex (Upper panel). Densitometry analysis of Western Blot is shown in the bottom panel. Data is represented as mean ± SEM (n=3 per genotype; **p<0.01; two tailed t-Test). Significance above bars represents comparison against WT.

**Figure S13. Protein co-expression modules in *Cul3^+/-^* cortex and cerebellum.** Hierarchical clustering of protein co-expression modules by module eigengene for *Cul3^+/-^* cortex and cerebellum. Module-genotype associations (*FDR<0.1) are shown below the dendrogram. **(a)** A total of 5 and 7 modules were significantly associated with *Cul3^+/-^* genotype in early postnatal and adult cortex, respectively. **(b)** A total of 8, 1, and 2 modules were significantly associated with *Cul3^+/-^* genotype in embryonic, early postnatal and adult cerebellum, respectively. Module enrichment analyses against literature-curated gene lists with previous evidence for involvement in autism are shown at the bottom (* FDR<0.05). **(c)** Transcriptome *vs* proteome correlation for cerebellum. DEGs for cerebellum from all developmental periods were overlapped with proteomes to extract corresponding proteins. The left panel shows Pearson’s correlation coefficient, each dot is one gene/protein, and Cul3 is highlighted in pink. Right panel represents GO term enrichment analysis of correlated genes/proteins.

**Figure S14. Cul3 impacts brain neurogenesis.** **(a)** Representative coronal sections of E14 brain stained for Ki67 (red) and DAPI (blue) in WT and *Cul3*^+/-^ mice (n = 4 mice for each genotype, two sections per animal). Scale bar is 100µm. The yellow boxes show the area selected for measuring cortical thickness. The right panel shows the quantification of cortical thickness. **(b)** The left panel shows the representative images of WT and *Cul3*^+/-^ E14 cortex stained for neuron progenitor marker Sox2 and DAPI. Scale bar 25µm; the quantification of %Sox2 positive cells in the section area shows no change in the right panel. **(c)** The left panel shows the representative image of WT and *Cul3*^+/-^ E14 cortex stained for cell cycle/proliferation markers Ki67 and DAPI; Scale bar 25µm, the quantification of %Ki67 positive cells in the section area shows no change in the right panel. **(d)** The left panel shows the representative images of WT and *Cul3*^+/-^ E14 cortex stained for post-mitotic neuron marker TBR1 and DAPI; Scale bar 25µm. The right panel shows the quantification of %TBR1 positive cells in the section area and demonstrates a significant reduction of the number of the newly generated neurons in Cul3*^+/-^* compared to WT (p<0.01, two tailed t-test).

**Figure S15**. **MEA recordings and phenotype rescue with Rhosin treatment**. **(a)** Representative single-well images containing primary cortical neurons surrounding electrodes from MEA plates for Rhosin-treated WT and *Cul3^+/-^*; scale bar is 100μm. **(b-c)**

Periodic MEA recordings and analyses of WT and *Cul3^+/-^* cortical neurons. The weighted mean firing rate and average burst frequency were measured for WT and *Cul3^+/-^* starting from day 4 to day 19. The reduction of both parameters was consistent throughout the recording sessions (n=7-13 mice for each genotype),*P<0.05, two tailed t-test. **(d-e)** The weighted mean firing rate and average burst frequency were measured after constitutive Rhosin treatment starting from day 4 to day 19. The Rhosin was able to rescue both parameters at day 8 (inset) but not at subsequent time points. *p<0.05, **p<0.01, ***p<0.001, ^#^p<0.05, ^##^p<0.01 (n=7-13 mice for each genotype); two tailed t-test; * represents comparisons to WT^VH^;  ^#^ represents comparisons to *Cul3^+/-^*^VH^.

**Figure S16. Venn diagrams for similarity of proteomics results our and two published *Cul3* conditional mouse models**. The comparison was carried out between nominally significant (without FDR correction) proteins identified in Dong et al. ^36^ and Rapanelli et al. ^37^ and our *Cul3^+/-^* FDR-significant proteins (FDR≤15%). None of the proteins identified in Dong or Rapanelli passed FDR significance cut-off, as pointed in the respective studies. **(a)** Comparison of our *Cul3^+/-^* mouse with Dong et al. Cul3^f/f^ Knockout and Cul3^f/+^ heterozygous mice. **(b)** Comparison of our *Cul3^+/-^* mouse with Rapanelli Cul3^f/+^ FC- forebrain specific and Cul3^f/+^ PFC Cul3^f/+^- prefrontal cortex.

**Figure S17. TPM values of CRISPR-off target genes detected by RNAseq**. Except Cul3, none of the genes is significantly altered in *Cul3^+/-^* mutant mice. Dots represent individual animals.

**Figure S18. Western Blot images for quantification of Cul3, total RhoA and active RhoA**. Western blot images used for quantification for adult cortex, hippocampus, cerebellum and embryonic cortex. The WB show Cul3 and total RhoA across different samples; GAPDH was used as loading control for Western Blotting normalization. Active RhoA was assayes for embryonic cortex. The immunoprecipitated active RhoA is shown; total RhoA, Cul3 and GAPDH across different samples are derived from the input samples.
